# Exploring anti-doping knowledge level: a systematic review among athletes, students, and athlete support personnel in the sports sector

**DOI:** 10.3389/fspor.2026.1778209

**Published:** 2026-06-03

**Authors:** Jorge Domínguez-Carrión, Arturo Casado, Rodrigo Pardo, Millán Aguilar-Navarro, Elena García-Grimau

**Affiliations:** 1 Departmento de Ciencias Biomédicas, Área de Educación Física y Deportiva, Faculty of Medicine and Health Sciences, University of Alcalá, Madrid, Spain; 2 Professional Worldwide Controls, Munich, Germany; 3 Research Center in Sports Science, Rey Juan Carlos University, Madrid, Spain; 4 Universidad Politécnica de Madrid, Madrid, Spain; 5 Faculty of Health Sciences, Institute of Health and Sport Sciences, Universidad Francisco de Vitoria, Madrid, Spain; 6 Faculty of Health Sciences, Universidad San Jorge, Zaragoza, Spain

**Keywords:** anti-doping, athlete support personnel, doping, education, knowledge, performance enhancement, sports

## Abstract

The level of anti-doping knowledge within the sports sector remains an underexplored area of research. Moreover, the methods used to assess this knowledge are not standardized, resulting in a lack of harmonization across studies. This heterogeneity makes it difficult to analyze and compare the level of knowledge among different populations in a consistent manner. The main aim of this study was to systematically review the level of anti-doping knowledge among Athletes, Students, and Athlete Support Personnel in the sports sector. The secondary objective was to identify and evaluate the methodology used to measure the level of knowledge. The *Preferred Reporting Items for Systematic Reviews and Meta-Analyses* (PRISMA) guideline was used to identify relevant studies. All articles assessing anti-doping knowledge were included, regardless of study design, measurement instruments, or target population. A set of evaluation criteria was developed and used to objectively measure the quality of the included studies. Ninety-three studies were included in the analysis. Twenty-five publications (26.88%) did not clearly report the level of anti-doping knowledge. Across the remaining studies, a wide heterogeneity of instruments and reporting approaches was observed. Using a heuristic and interpretive classification framework to synthesize author-reported descriptors, most studies were heuristically classified as reflecting a predominantly “Low” level of anti-doping knowledge (45.16%), while fewer studies reported “Limited” (8.60%), “Basic” (5.38%), or “Good” (13.98%) knowledge. No study explicitly described an “Extensive” level of anti-doping knowledge. These findings should be interpreted as indicative patterns derived from descriptive synthesis rather than as precise quantitative estimates. Despite methodological heterogeneity, these findings highlight the need to develop standardized and validated assessment tools and to implement structured educational programs to improve anti-doping knowledge across the sport sector.

**Systematic Review Registration:**

https://www.crd.york.ac.uk/PROSPERO/view/CRD42024513234, identifier [CRD42024513234].

## Introduction

The first international text on doping in sports was Resolution 67/12 of the Council of Europe, which defined doping as:

Administration to or the use by a healthy person, in any manner whatsoever, of agents foreign to the organism, or physiological substances in excessive quantities or introduced by an abnormal channel, with the sole purpose of artificially affecting and by unfair means the performance of such a person when taking part in a competition (1).

The World Anti-Doping Agency (WADA) was established in 1999 as an international independent agency to lead a collaborative worldwide movement for doping-free sport. Its main objective was to develop, harmonize, monitor, and coordinate the fight against doping in sports by establishing and enforcing strict guidelines and rules. In this way, the doping definition was also universalized, considering the phenomenon of doping not only as an unethical action to enhance performance in sports by using prohibited substances or external aid, but as the occurrence of one or more of the Anti-Doping Rule Violations (ADRVs) outlined in the World Anti-Doping Code (WADC). The WADC is the core document that standardizes anti-doping policies across sports organizations and public authorities worldwide (2). The first version, published in 2003, recognized only eight ADRVs. Currently, the 2021 edition incorporates eleven ADRVs, as outlined in Article 2.1 to 2.11 (3). Understanding what doping is, is a crucial step toward developing efficient prevention programs (4) that can help mitigate the associated risks in various sporting contexts. Therefore, it is important to implement prevention strategies and conduct a comprehensive assessment of target populations to understand better their knowledge and attitudes toward doping (5). Given the potential deterrent effect that Athletes' and Athlete Support Personnel's (ASP) knowledge can have on anti-doping compliance and prevention — due to awareness of the possible serious consequences of regulatory violations (6) — this study seeks to identify and synthesize previous research in this field before determining the most appropriate actions and interventions. Athletes and ASP must also recognize that “not knowing about the rules and regulations can lead to an inadvertent ADRV” (6). This is directly related to the fundamental principle underlying the WADC: Strict liability (2). In the case of an ADRV being committed, the strict liability principle is imposed, regardless of whether the violation was committed intentionally or not (7). Consequently, the individual is held accountable for the violation irrespective of their intentions. It is crucial to ensure compliance with ADRVs to avoid any potential legal or financial sanctions.

Education as a prevention strategy is a cornerstone of the fight against doping. It has recently been certified by the implementation of the International Standard for Education (ISE) (8). The goal of education in this context is to foster a culture of clean sport, uphold its core values, and prevent the use of Performance-Enhancing Substances (PESs) among Athletes and other individuals. In this sense, it is essential that Athletes receive anti-doping education before being subjected to doping control, although it is also the ASP's responsibility to be “knowledgeable of, and comply with all anti-doping policies and rules” (9). Given the inherent nature of sports, the constraints on resources for doping controls, and the athlete-centered approach adopted by most anti-doping organizations, International Federations and WADA educational initiatives have primarily targeted elite-level Athletes (7). Moreover, most social science research on doping in sports has focused on Athletes (10), while there appears to be a scarcity of literature that discusses the attitudes, beliefs, and knowledge of ASP (11). Accordingly, there seems to be a lack of comprehensive qualitative and quantitative instruments capable of encompassing all anti-doping rules in a manner comparable to the WADC (7).

Despite the increasing number of studies addressing anti-doping education, the concept of anti-doping knowledge has frequently been used inconsistently across the literature. In many cases, instruments described as assessing knowledge also include items related to awareness, attitudes, beliefs, perceptions, or moral evaluations toward doping. Although these constructs are interrelated and may jointly influence behavior, they are analytically distinct. The lack of conceptual clarity regarding what constitutes anti-doping knowledge contributes to substantial heterogeneity in study designs, assessment tools, and reported outcomes, thereby limiting the comparability and interpretability of existing findings.

In the present review, anti-doping knowledge is conceptualized as objective, factual knowledge of the anti-doping system, including understanding of the World Anti-Doping Code and its core principles (e.g., strict liability), ADRVs, prohibited substances and methods, doping control procedures, and the rights, responsibilities, and sanctions applicable to Athletes and Athlete Support Personnel. This definition intentionally distinguishes knowledge from subjective constructs such as attitudes, beliefs, or perceptions. As discussed later in the Limitations section, many of the included studies do not make this distinction explicit, often combining multiple constructs within a single assessment. Recognizing this conceptual overlap is essential for accurately interpreting the evidence base and underscores the need for standardized and validated tools that isolate anti-doping knowledge as an independent construct.

Given the absence of standardized studies on anti-doping knowledge and the lack of consistent reporting using standardized instruments to measure it, we believe in the importance of conducting a thorough and comprehensive review to identify all existing research assessing the anti-doping knowledge across different populations. Furthermore, it is important to compare the level of anti-doping knowledge among different populations (Athletes, Students, and ASP); across different levels of sports practice (recreational, amateur, and professional); on different topics (rights and responsibilities, prohibited substances and methods, rules and regulations, doping control procedures, ADRVs, nutritional supplements and external aid, side effects, and sanctions); and between different cultures and/or regions.

To the best of the author's knowledge, no previous systematic review has comprehensively analyzed the level of anti-doping knowledge. Therefore, there is a clear gap in the literature regarding studies that assess and compare this knowledge across populations. The main objective of this study was (1) to conduct a systematic review of published studies that analyze the level of anti-doping knowledge and its distribution across publication periods among different Athletes, Students, and Athlete Support Personnel (ASP) who are or will become part of the sports sector. Additionally, this research aimed to achieve specific objectives: (2) to evaluate the quality of the included studies; (3) to determine the methodology used to measure the level of anti-doping knowledge; and (4) to identify the most relevant authors and countries, as well as the number of publications conducted in this field of study over time.

## Methodology

The systematic review was conducted following the PRISMA recommendations (12) as a guideline for study identification, screening, eligibility, and inclusion. This research was registered with the International Prospective Register of Systematic Review (PROSPERO), with the registration number CRD42024513234.

### Search strategy

A comprehensive literature search was conducted on 20 January 2024 in PubMed, Scopus, SPORTDiscus, and Web of Science Core Collection databases, including all records indexed up to 31 December 2023 (*n* = 440). The search was limited to the consideration of the search terms in the Title, Abstract, and Keywords fields using structured Boolean logic with explicit parentheses to ensure reproducibility. Limiting searches to these indexed fields was intended to enhance specificity; however, this approach may have reduced sensitivity by omitting relevant studies that did not explicitly use the selected terminology in searchable metadata. In addition, because the search strategy required the inclusion of education-related terminology (e.g., “educa*”, “formati*”, “training”), studies assessing anti-doping knowledge that did not explicitly frame their work within an educational context may not have been captured. The electronic search strategy combined controlled Boolean operators across four predefined conceptual components: (1) doping-related terminology (“doping” OR “anti-doping” OR “antidoping”); (2) sport-related context (“sport*” OR “physical activity” OR “physical education”); (3) educational dimension (“educa*” OR “formati*” OR “training”); and (4) knowledge (“knowledge”). These components were systematically combined to maximise retrieval sensitivity while maintaining thematic specificity. Truncation symbols (e.g., sport*, educa*, formati*) were used where supported to capture lexical variations. In operational terms, the following Boolean combinations illustrate the conceptual structure of the search strategy used to identify the thematic content of relevant studies: “anti-doping AND knowledge AND sport AND education”, “antidoping AND knowledge AND sport AND education”, “doping AND knowledge AND sport AND education”, and “(anti-doping OR antidoping OR doping) AND knowledge AND sport AND education”. The exact database-specific search strings used in each database, including field tags and syntax adjustments, are provided in Supplementary Appendix A to ensure full reproducibility. Searches were performed in database-specific indexed fields: “Topic” (Web of Science), “Title-Abstract-Keywords” (Scopus), “Title-Abstract” (SPORTDiscus), and “Title-Abstract” (PubMed).

The full search strings for each database, including field tags and controlled vocabulary (e.g., MeSH terms in PubMed where applicable), are provided in Supplementary Appendix A.

Search results from all used databases were exported to EndNoteTM X9 (Philadelphia, United States of America) software. After this, all files were managed individually in this tool identifying duplicates automatically using “Find Duplicates” functionality. A total number of 126 articles were automatically identified and removed. Any additional persisting duplicates (*n* = 42) were manually removed during the identification process. A total number of two-hundred seventy-two (*n* = 272) articles were remaining.

### Exclusion criteria and study selection

Forty-eight studies were excluded as they did not meet the established eligibility criteria of (1) being a systematic review or review (*n* = 48). Additionally, (2) articles not written in English were also excluded (*n* = 40), but in other languages such as Croatian (*n* = 1), French (*n* = 5), German (*n* = 6), Italian (*n* = 5), Japanese (*n* = 3), Polish (*n* = 7), Portuguese (*n* = 3), Russian (*n* = 2), Slovakian (*n* = 1), Spanish (*n* = 6) and Turkish (*n* = 1). The restriction to English-language publications was applied due to feasibility constraints and resource limitations for translation. However, this may introduce language bias, as relevant studies published in other languages could have been excluded. Given that anti-doping education research is conducted globally, particularly in non-English-speaking countries, this restriction may lead to an underrepresentation of certain regional perspectives and potentially overrepresent findings from English-dominant academic contexts. After analyzing the (3) title and abstract to identify content mismatching related to the field of study, one hundred-three records were excluded (*n* = 103). In case these sections were deemed eligible, the entire full-text was analyzed.

At this point, eighty-one studies were included in the study (*n* = 81). After analyzing the full text in detail, ten studies were discarded because their content did not align with the aim of this investigation (*n* = 10). At this point, seventy-one studies were included in the study (*n* = 71). Additionally, a manual search was performed by reading through the references section of the retrieved and selected studies to identify similar results that fulfilled the eligibility criteria (*n* = 22). Finally, a total number of ninety-three studies were included in this review (*n* = 93).

During the eligibility process, studies focusing on Students (e.g., from school, high school, and university level) were selected (*n* = 17), and articles studying Athletes and/or Para-Athletes were included (*n* = 49). Other investigations pointing to ASP (Health Professionals and Non-Health Professionals) were included (*n* = 36). This comprehensive approach ensures a diverse representation within our research. These populations were selected to represent the primary stakeholders involved in the sports sector ecosystem, including both direct participants (Athletes) and supporting or future professionals (Students and ASP).

**Figure 1:**
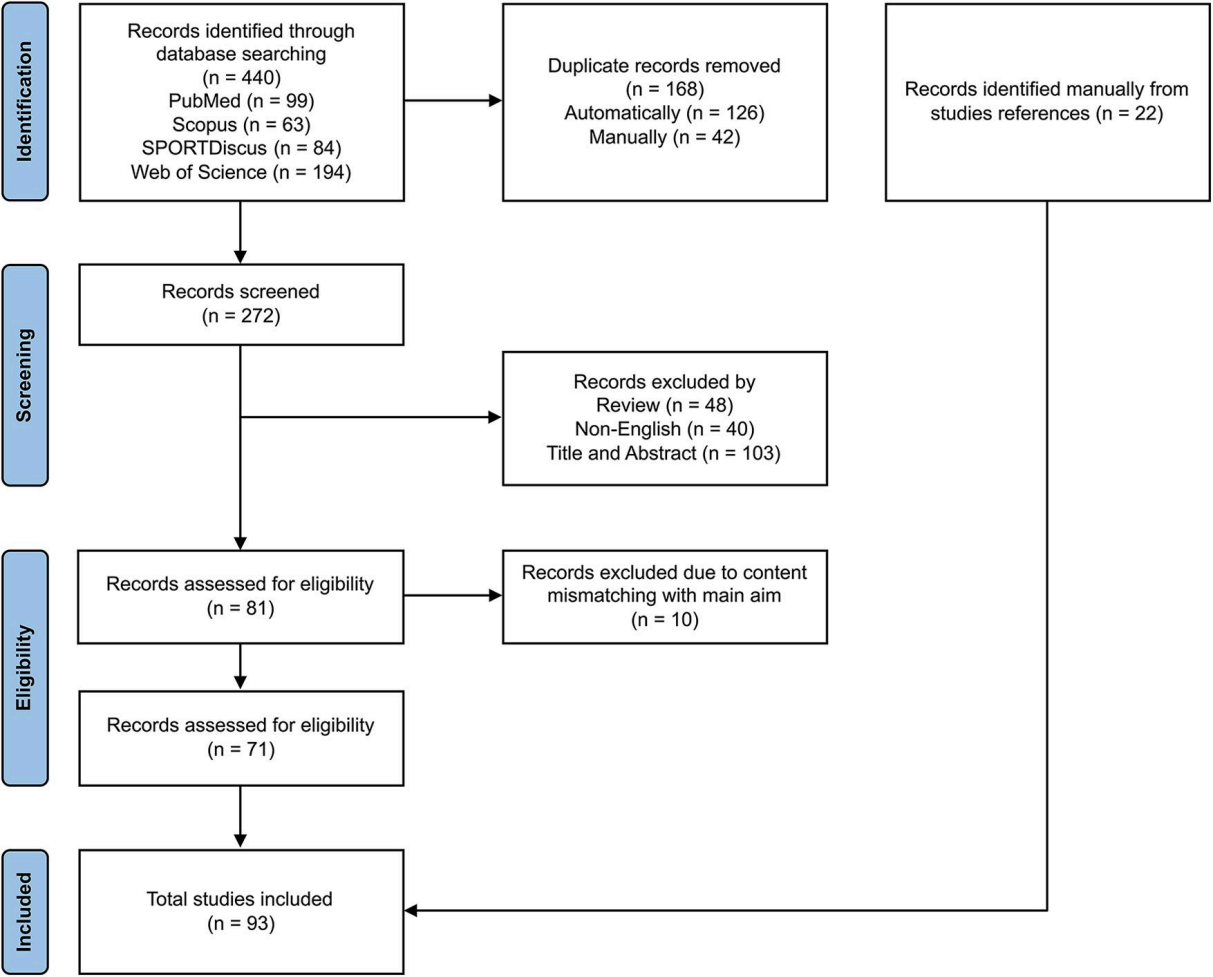
PRISMA 2020 flow diagram of the study selection process. The diagram summarizes identification, screening, eligibility, and inclusion stages, including searches of databases, registers, and additional sources conducted up to December 2023.

Lastly, studies were considered eligible if they employed quantitative, qualitative, or mixed-methods designs and if they were published in a peer-reviewed English-language journal. Accordingly, non-peer-reviewed publications, conference papers, books, and/or book chapters were not included in this research. Title and Abstract screening were conducted independently by two reviewers (JDC and RP). Full-text eligibility assessment was also performed independently. Disagreements at either stage were resolved through discussion until consensus was reached. The methodology employed in this study was consistent with the PRISMA guidelines to ensure the comprehensive inclusion of all relevant studies, as depicted in Figure 1.

### Quality assessment list

The methodological reporting quality of the included studies was evaluated using a checklist adapted from the Consolidated Standards of Reporting Trials (CONSORT) (13) and the Strengthening the Reporting of Observational Studies in Epidemiology (STROBE) guidelines (14). The methodologies outlined have been previously employed in various studies, notably including the research conducted by (15). These frameworks were used to assess key reporting and methodological transparency indicators across the included studies. Because the included studies comprised heterogeneous observational designs and descriptive surveys, a formal risk-of-bias tool was not applied; instead, reporting transparency and methodological clarity were assessed using adapted CONSORT and STROBE criteria. Accordingly, the quality appraisal relied exclusively on methodological reporting indicators derived from adapted CONSORT and STROBE criteria.

The checklist was designed to capture key methodological transparency and reporting indicators across the included studies, based on the following criteria: (1) anti-doping knowledge assessment identified separately in the aim(s); (2) descriptive data of participants identified; (3) anti-doping knowledge level clearly defined; (4) methodology description detail; (5) limitations clearly identified; (6) instruments validation performed; and (7) study interventions number. The number of participants was also considered for inclusion in this list. However, the type of participants is variable, and it would not be possible to estimate an appropriate sample size. In addition to that, all items were rated individually from “0” to “2”, as shown in Supplementary Table 1 (see Supplementary Material).

The quality of the studies was determined by calculating a raw total score ranging between “0” and “14”. Afterwards, each raw score was converted to a standardized scale ranging from 0 to 10, with higher scores indicating better quality. Studies were then classified into three quality tiers based on tertiles of the standardized score. Those with a standardized score of ≥ 6.429 were included in “Tertile 1 (T1)”, studies scoring between 5.000 and 6.428 were categorized as “Tertile 2 (T2)”, and those scoring ≤ 4.999 were classified in “Tertile 3 (T3)”. Tertile thresholds were calculated based on the distribution of standardized quality scores across the included studies to allow transparent and reproducible classification. The quality assessment was independently conducted by two reviewers (JDC and RP). Any discrepancies were discussed in a consensus meeting until agreement was reached.

The standardized aggregated score reflects a combination of methodological transparency and reporting completeness. It should not be interpreted as a formal measure of risk of bias but rather as a structured overview of study quality characteristics.

The quality appraisal was used exclusively to provide an overview of methodological reporting characteristics of the included studies. The synthesis and interpretation of findings were not weighted or stratified according to the aggregated quality score.

### Knowledge level coding procedure

A predefined framework was established prior to data extraction to classify reported knowledge levels into five categories (Low = 1; Limited = 2; Basic = 3; Good = 4; Extensive = 5) based on author-provided descriptors. When numerical scores were reported without a qualitative label, classification was based on thresholds or scale interpretation described in the study; if none were provided, categorization was derived proportionally according to the scoring range and contextual interpretation. In studies reporting multiple subdomains, an overall category was assigned according to the predominant interpretation, or conservatively when discrepancies were present. Coding was performed independently by two reviewers (JDC and RP), and discrepancies were resolved through discussion until agreement was reached.

To ensure internal consistency, all extracted data were re-checked prior to final analysis. Frequencies, percentages, and category assignments were recalculated based on the final dataset of included studies (*n* = 93). Numerical coding of knowledge levels was systematically aligned with the predefined five-point heuristic framework (Low = 1; Limited = 2; Basic = 3; Good = 4; Extensive = 5), and all figures and tables were updated accordingly.

## Results

### Methodological design of the studies

In line with the aims of this systematic review, only a small proportion of the included studies (*n* = 4) adopted a longitudinal research design (16–19). The vast majority (*n* = 89) used a cross-sectional design to assess the level of anti-doping knowledge in different populations.

The questionnaire (*n* = 83) emerged as the most frequently employed instrument for data collection among quantitative and/or mixed-method designs, whereas interviews (*n* = 9) and telephone inquiry (*n* = 1) were less commonly used among qualitative and/or mixed-method designs.

### Leading authors and countries

The included publications were conducted by 82 different leading authors. The biggest number of studies (*n* = 5) was conducted by Cornelia Blank (Austria), followed by Yuka Murofushi (Japan) with three publications. Other authors who led two studies (*n* = 2), including anti-doping knowledge assessment, were Dušan Antić (Serbia), Terry Engelberg (Australia), Jaime Morente-Sánchez (Spain), David Mottram (UK), and Kathrin Weber (Austria).

To determine the country of origin for each publication, data was collected based on the first author's affiliation. This publication comprises studies conducted in a total of forty-four countries. Only three countries [Austria (*n* = 8); Turkey (*n* = 8); and Australia (*n* = 6)] conducted more than five studies, representing 23.66% of the total studies. A total of four studies (17.20%) were performed by four different countries, while there were four countries where three publications (12.90%) were made. Ten additional countries published a total of two studies (21.51%), and the rest of the countries (*n* = 23) only conducted a single publication, representing 24.73% of the total. A detailed country-level distribution of the included studies is provided in Supplementary Table 2 (see Supplementary Material).

### Publication year

**Figure 2:**
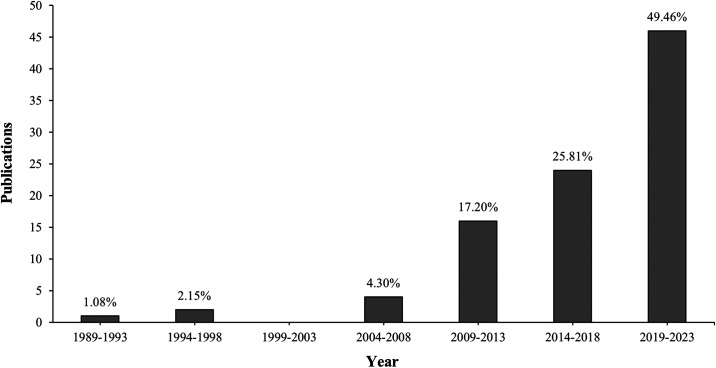
Distribution of included publications across five-year publication windows (1989–2023). Values represent the percentage of studies published within each aggregated time period included in the review. These data describe patterns in the publication record and do not reflect longitudinal changes in anti-doping knowledge within populations.

Figure 2 illustrates an increase in the number of publications over the past fifteen years. Before 2009, only seven studies (7.53%) assessing anti-doping knowledge were identified, whereas between 2009 and 2013, sixteen studies (17.20%) were published, representing an increase compared to the previous period. From 2014 to 2018, publications kept increasing with 24 studies (25.81%). In the last 5 years (2019–2023), the number of studies published was forty-six (49.46%). The articles published, including anti-doping assessment, reached their peak in 2022 (*n* = 13).

### Anti-doping knowledge level

Of the overall studies, twenty-five publications (26.88%) did not clearly report the level of knowledge of the studied population. Percentages were calculated based on the total number of included studies (*n* = 93), including those that did not clearly report a defined knowledge level.

The most frequently reported level of knowledge was “Low”, observed in forty-two studies (45.16%). In contrast, eight publications (8.60%) reported a “Limited” level of knowledge, whereas only five studies (5.38%) indicated a “Basic” level of knowledge. In this review, thirteen studies (13.98%) reported a “Good” level of knowledge, while no publication identified an “Extensive” level. These distributions should be interpreted as indicative patterns emerging from qualitative synthesis rather than as precise quantitative estimates of anti-doping knowledge. Category assignment followed the predefined coding procedure described in the Methodology section.

Given the heterogeneity of instruments, constructs, and descriptors used across the included studies, the classification of anti-doping knowledge into five levels (Low, Limited, Basic, Good, and Extensive) should be understood as a heuristic and interpretive framework rather than a definitive or standardized taxonomy. This categorization was developed to facilitate a structured synthesis of a fragmented literature in which authors used diverse and often subjective descriptors to characterize knowledge levels. Accordingly, these levels do not represent equivalent psychometric thresholds across studies, nor should they be interpreted as directly comparable quantitative measures. Each level of knowledge was assigned a numerical value on a five-point scale: Low (identified as Deficit; Inadequate; Insufficient; Lack of knowledge; Low; Poor) = 1; Limited (identified as Limited; Not enough) = 2; Basic (identified as Basic; Fair; Reasonable) = 3; Good (identified as Adequate; Good; Moderate; Satisfactory; Suboptimal) = 4; Extensive (not identified in any of the studies) = 5. A higher value indicates a higher level of knowledge.

**Figure 3:**
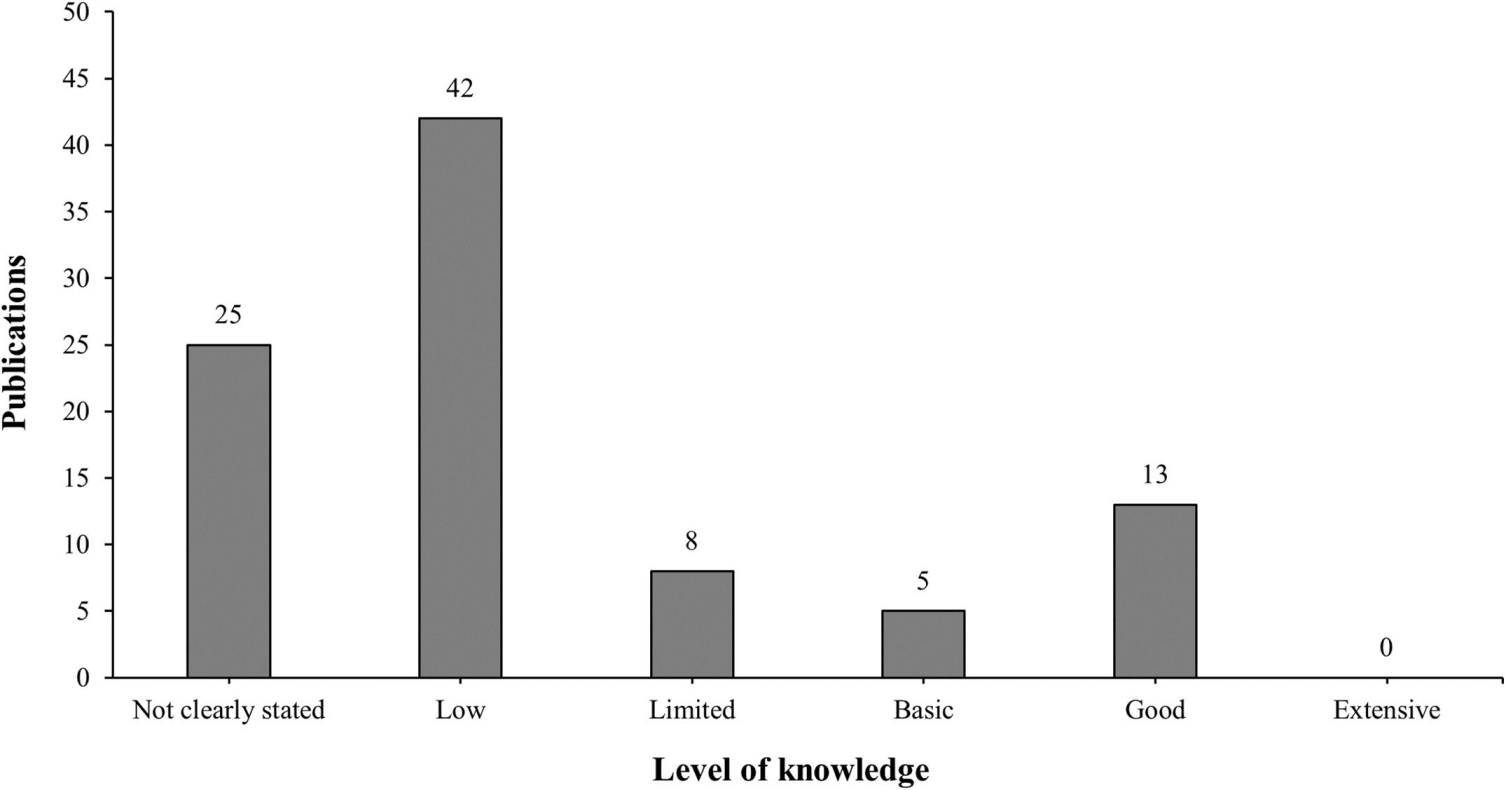
Distribution of reported anti-doping knowledge levels across included studies, based on heuristic categorization of heterogeneous study outcomes. Values represent aggregated classifications derived from independent cross-sectional studies and reflect descriptive patterns in the published literature. These data do not imply equivalent measurement thresholds across instruments and should not be interpreted inferentially.

Numerical values were used exclusively for descriptive aggregation and visualization. They do not imply equal intervals or statistically meaningful differences and should not be interpreted inferentially. The distribution of reported anti-doping knowledge levels is illustrated in Figure 3.

### Temporal patterns in reported anti-doping knowledge levels across publication periods

From 1994 to 1998, the level of anti-doping knowledge remained at a “Low (1)” level, with only one study (20) identifying that measured the level of anti-doping knowledge clearly. Furthermore, between the periods 1989–1993 and 1999–2003, no studies were found that satisfied this condition. Between 2004 and 2008, the highest level of knowledge (2.50) was reached, with results between the “Limited (2)” and “Basic (3)” levels. A total of four publications (21–24) reported these results. Publications from the period 2009–2013 most frequently reported knowledge levels slightly below the “Limited (2)” classification, based on ten studies. Across subsequent publication periods, studies reported similar knowledge classifications based on data obtained from eighteen publications between 2014 and 2018; and after analyzing the results reported by the thirty-five studies carried out between 2019 and 2023. The aggregated distribution of reported knowledge classifications across publication periods is presented in Supplementary Figure 4 (see Supplementary Material).

### Athletes' anti-doping knowledge

Fourteen studies did not clearly report the level of anti-doping knowledge in Athletes (16, 18, 25–36). Detailed methodological characteristics are presented in Supplementary Table 3 (see Supplementary Material), which presents the data collected from various studies included in this systematic review, and highlights the level of anti-doping knowledge among Athletes.

More recent publications more frequently report “Basic” to “Low” levels of anti-doping knowledge among Athletes. Given that these data originate from independent cross-sectional studies, this pattern should be interpreted descriptively rather than as evidence of a true decline over time. Across the included publications since the first study in 2007 (22), reported classifications range from “Basic (3)” to between “Low (1)” and “Limited (2)” levels in studies published between 2019 and 2023. Supplementary Figure 5 presents levels of anti-doping knowledge among Athletes (see Supplementary Material).

### Students' anti-doping knowledge

Five studies did not clearly report the level of anti-doping knowledge in Students (17, 19, 31, 37, 38). Detailed study characteristics are provided in Supplementary Table 4.

The overall data of the studies that assess Students' level of anti-doping knowledge in this systematic review are depicted in Supplementary Table 4.

Most of the research [82.35% (*n* = 14)] was conducted in a university-level educational context. The sample was similarly distributed between the study disciplines of health and sports.

Later publications more frequently report “Limited” to “Basic” levels of anti-doping knowledge among Students. Since the first study published in 2007 (24), later publications more frequently classify knowledge between “Limited (2)” and “Basic (3)” levels. Because these findings derive from independent cross-sectional studies conducted in different contexts, this pattern should be interpreted descriptively rather than as evidence of a temporal increase in knowledge within the same student populations. Supplementary Figure 6 presents levels of anti-doping knowledge among Students (see Supplementary Material).

### Athlete support personnel anti-doping knowledge

Supplementary Table 5 depicts the overall data of the studies of Health professionals ASP level of anti-doping knowledge of this systematic review. Supplementary Table 6 depicts the overall data of the studies of Non-Health professionals ASP level of anti-doping knowledge of this systematic review.

#### Health professionals

Five studies did not clearly report the level of anti-doping knowledge in health professional ASP (34, 39–42). Study characteristics are summarized in Supplementary Table 5 (see Supplementary Material).

In this study, general practitioners accounted for 39.13% (*n* = 9) of the studies, reporting a low level of anti-doping knowledge in all cases. On the other hand, pharmacists are the most representative sample within this population [47.83% (*n* = 11)]. However, general knowledge is also low.

The level of anti-doping knowledge of Health professionals ASP has remained between the levels “Low (1)” and “Limited (2)” over the years. Since the first study was published in 1997 (20), reported classifications have remained predominantly between “Low (1)” and “Limited (2)”, with minor variation across publication periods. Supplementary Figure 7 presents levels of anti-doping knowledge among Health professionals ASP (see Supplementary Material).

#### Non-health professionals

This review reflects the importance of the role of the coach, as they represent 75.00% (*n* = 12) of the sample size of non-health professionals in the selected studies. Compared with other populations, coaches appear to demonstrate relatively higher classifications within the “Basic (3)” range; however, this difference should be interpreted cautiously, given the heterogeneity of instruments and study designs.

Six studies did not clearly report the level of anti-doping knowledge in non-health professional ASP (5, 16, 25, 34, 43, 44). Detailed information is provided in Supplementary Table 6 (see Supplementary Material).

Published studies consistently classify knowledge levels among non-health professionals within a similar categorical range across publication periods.

Since the first study published in 2006 (23), the level of knowledge has remained at “Basic (3)”, with a slight decrease in the level of knowledge between “Limited (2)” and “Basic (3)” in the period from 2019 to 2023. These findings reflect descriptive patterns in the literature rather than longitudinal changes within the same populations. Supplementary Figure 8 presents levels of anti-doping knowledge among Non-Health professionals ASP (see Supplementary Material).

## Discussion

The purpose of this review was to systematically identify and synthesize a comprehensive range of published studies examining anti-doping knowledge among different populations who are, or will become, part of the sports sector. In addition, our analysis aims to evaluate the overall level of anti-doping knowledge, identify the methodologies employed to measure it, and classify the knowledge level across different demographic groups. A total of ninety-three studies met the inclusion and exclusion criteria. To the best of our knowledge, this is the first systematic review to provide such an extensive analysis accompanied by a quality assessment of the included studies.

Temporal patterns reflect aggregated findings from independent cross-sectional studies and should not be interpreted as longitudinal changes within populations.

It must be acknowledged that the currently available evidence does not provide a clear picture of the level of knowledge that has been attained concerning anti-doping. However, the findings indicate a widespread lack of anti-doping knowledge in the different groups analyzed. The diversity of methodologies, populations, and data collection instruments, together with the considerable variability in the study results and inherent limitations, undermines the overall credibility and comparability of the available data. Consequently, it is imperative to exercise prudence when attempting to extrapolate the findings of individual studies to a broader context. The quality appraisal was based exclusively on methodological reporting criteria derived from adapted CONSORT and STROBE indicators. Importantly, this appraisal was intended to describe reporting characteristics rather than to determine the weight of individual studies in the narrative synthesis. When examining the limitations identified through this evidence synthesis, a clearer and more structured pathway has emerged for researchers seeking to generate more robust and standardized data on anti-doping knowledge. As such, this provides a valuable opportunity to enhance the accuracy and reliability of future research in this field. These findings should be interpreted in light of the conceptual distinction adopted in this review between objective anti-doping knowledge and related constructs such as attitudes, beliefs, or awareness, as the frequent lack of separation between these domains in the existing literature represents a central methodological challenge.

The main findings of this review showed a lack of anti-doping knowledge among all populations examined in the selected studies, with the most common level of knowledge identified as “Low”. When interpreted in light of the strict liability principle underpinning the World Anti-Doping Code, these findings raise important normative and regulatory considerations. Strict liability assigns full responsibility for Anti-Doping Rule Violations regardless of intent or level of understanding, which may be perceived as unreasonable or unjust in contexts where anti-doping knowledge is demonstrably insufficient. In this sense, limited knowledge constitutes not only an educational shortcoming but also a structural risk factor for inadvertent violations. Therefore, implementing well-designed educational interventions is necessary (45), and the inclusion of specific anti-doping topics within formal education curricula could enhance regulatory fairness while ultimately benefiting Athletes (39).

This tension reinforces the ethical obligation of Anti-Doping Organizations and educational institutions to align the enforcement of strict liability with proportionate and effective educational provision.

### Athletes

In this review, the main findings show a low level of general knowledge on anti-doping, and the ease of delegating responsibilities to a third party, such as the task of registering whereabouts in ADAMS (and/or any updates related to it), may contribute to a lack of personal engagement with this topic (46). Moreover, the use of different knowledge assessment tools among Athletes makes it difficult not only to compare results between different studies but also to obtain a clear outcome. This inconsistency hinders cross-study comparisons (47), not only within the same population but also to compare among other existing stakeholders in the field.

The majority of studies used questionnaires to measure knowledge level, but the content widely differed among them. All studies were cross-sectional in design, which suggests a need to implement longitudinal studies to collect more reliable data. Furthermore, several studies published in more recent periods report predominantly “Low” to “Basic” knowledge levels (27, 32, 48–55); however, substantial heterogeneity in study design, cultural context, and knowledge assessment instruments limits cross-study comparability and restricts the generalization of these findings.

### Students

According to (8), Sport Sciences graduates need to understand the negative effects of doping on Athletes' health, although they do not need to be legal experts in anti-doping regulations. At the very least, future sports professionals may know the basics of anti-doping to be able to support Athletes and encourage them to achieve the highest performance level while respecting and adhering to the rules. The current review states that the level of anti-doping knowledge among Students is predominantly low, as reported in the majority of studies (*n* = 7). In line with this finding, previous research has also shown that university Students preparing to become teachers and coaches demonstrate limited knowledge about nutrition and its impact on performance (56). However, several more recent studies report “Limited” to “Basic” levels of anti-doping knowledge among Students, which may reflect increasing attention to doping prevention initiatives and awareness of the harmful consequences associated with performance-enhancing substances (57).

The findings of this study suggest that there is a need to design standardized, mandatory educational programs specifically tailored to the needs of Students. Based on these results, the authors recommend the implementation of such programs as a part of the university curriculum in order to achieve the desired improvement in anti-doping knowledge. Although previous studies have shown positive effects of educational interventions in specific student populations (7, 8, 17, 19, 38, 58–60), differences in educational settings, cultural backgrounds, and measurement approaches across studies limit cross-study comparisons and preclude generalized conclusions.

### Athlete support personnel

#### Health professionals

The findings indicate that doctors lack adequate knowledge regarding doping agents. Consequently, they may provide inaccurate advice when consulted by Athletes (61). This can endanger not only the health of the Athlete but also jeopardizes their sporting careers by increasing the risk of an unintentional ADRV. Beyond athlete-related consequences, insufficient anti-doping knowledge among medical staff raises important issues of professional responsibility, as inaccurate or incomplete advice may directly contribute to regulatory violations under the principle of strict liability. In this context, misinformation provided by Health professionals may also have ethical and legal implications, given their duty of care and their recognized role as trusted advisors in Athletes' health and medication management. Therefore, medical professionals must receive adequate information and training on doping agents to ensure accurate and effective advice to Athletes, while also understanding the professional, ethical, and regulatory risks to which they may be exposing both Athletes and themselves (62). On the other hand, Pharmacists are the most representative sample within this population [47.83% (*n* = 11)], however, their overall level of anti-doping knowledge was also reported as low. In addition, nutritionists were not part of the sample of any included study.

Across publication periods, studies consistently classify knowledge levels between “Low” and “Basic”, although these findings represent descriptive patterns in the literature rather than longitudinal changes within the same populations. This may be attributed to a general lack of awareness of anti-doping regulations and/or the absence of this topic within their formal educational curricula (63).

While the included studies indicate a need for anti-doping-specific educational programs among healthcare professionals (64–66), variation in professional roles, national anti-doping frameworks, and assessment instruments limits the comparability of findings and constrains broader generalization.

#### Non-health professionals

According to the definition of Athlete Support Personnel, any individual involved in the provision of care, treatment, or assistance to an Athlete taking part in a sports competition (3) may have an impact on the person. Among ASP, coaches appear to be the main influence and source of information for Athletes (4). This review reflects the importance of this role, as coaches represented 75.00% (*n* = 12) of the total sample in the studies analyzed. Compared with other groups, coaches apparently have slightly greater knowledge about anti-doping matters. However, these results suggest the need for further research focused on coaches, providing them with greater support and implementing mandatory anti-doping education at all stages of their professional training and career (10).

Across the included studies, the level of knowledge among coaches has remained predominantly within the “Basic (3)” range, with slight variation between “Limited (2)” and “Basic (3)” across publication periods, consistent with the descriptive patterns reported in the Results section; however, it is recommended to maintain ongoing training to improve their knowledge in the future (48). Furthermore, although existing evidence suggests recognition of coaches' responsibility in doping prevention and a desire for systematic anti-doping education (11), heterogeneity in participant profiles, cultural contexts, and methodological approaches limits direct comparison and warrants caution when drawing generalized conclusions.

## Limitations

The results obtained may not be generalized due to the lack of consistency in the instruments used in the existing studies to measure the anti-doping knowledge. Quantifying the overall level of knowledge was not possible due to the lack of standardized quantitative tools, which directly weakens the internal comparability of studies and limits the strength and external applicability of the synthesized evidence. Moreover, many of the included investigations combined attitudes, beliefs, perceptions, and awareness with what was labeled as anti-doping knowledge, rather than assessing anti-doping knowledge as an independent construct defined by objective, factual understanding of rules, procedures, and Anti-Doping Rule Violations, thereby increasing conceptual ambiguity and further limiting the interpretability of the findings. In addition, study designs were predominantly cross-sectional, analyzing data from a population at a single point in time, which limits the ability to infer trends over time. This limitation is particularly relevant for the interpretation of time-related findings, as the sparseness of publications in earlier periods and the reliance on independent cross-sectional samples preclude valid inferences about longitudinal trends in anti-doping knowledge. Another limitation was the heterogeneity of topics covered across the studies, which varied considerably and included, among others: the concept of doping, what WADA is, sources of information, prohibited substances and methods, dietary supplements, side effects, doping control procedures, anti-doping rule violations, sanctions and consequences, and rules and regulations. Hence, it was not possible to compare results between different studies or populations, which reduces the robustness of cross-study comparisons and the generalizability of the reported knowledge levels. In addition, the size of the sample was not consistent across investigations.

Moreover, the five-level classification of anti-doping knowledge adopted in this review is inherently heuristic. Although a predefined and independently applied coding framework was used to enhance consistency, synthesis across non-homogeneous instruments and author-defined descriptors necessarily involves interpretative judgment, limiting strict comparability and generalization.

Finally, although we used widely recognized search strategies to identify relevant papers, some articles may have been unintentionally overlooked. This could be due to the selection of keywords used in the search engine or because they were not referenced in the journal articles cited in the systematic review. Consequently, such omissions could affect the completeness of the dataset. In particular, the inclusion of education-related terms within the search strategy may have limited sensitivity by excluding studies assessing anti-doping knowledge that did not explicitly frame their work within an educational or training context.

Additionally, the exclusion of non-English publications may have contributed to language bias, potentially limiting the geographic and cultural diversity of the evidence base. This may affect the generalizability of findings, particularly in regions where anti-doping education research is disseminated primarily in local languages.

Importantly, the high degree of methodological, cultural, and conceptual heterogeneity across studies precludes robust synthesis or generalization of results. Differences in how anti-doping knowledge was defined, operationalized, and measured restrict the comparability of findings and limit the strength of any overarching conclusions drawn from this review.

## Future research

Gaining a comprehensive understanding of this matter may require additional analysis and investigation. Including longitudinal studies and interventions will help to determine the real level of anti-doping knowledge of any population. Before this, the design and validation of a standardized questionnaire worldwide would be necessary to be able to obtain accurate results and compare them among different sample populations. In addition, conducting assessments of the Athlete's parents would enrich the quality of the information since only one study was found targeting this type of population as an essential part of the ASP.

## Practical applications

To effectively measure the level of anti-doping knowledge, researchers need to develop a tailored and validated questionnaire that covers all relevant aspects of anti-doping knowledge. This is essential to accurately quantify the level of knowledge, ensuring results can be compared across different populations universally. Developing a standardized questionnaire will also help to harmonize the way the results are measured and interpreted.

## Conclusions

To the best of the authors' knowledge, this study represents the first systematic review to present the level of anti-doping knowledge among Athletes, Students, and Athlete Support Personnel within the sports sector. Although the diversity of methods and instruments employed in the included studies limits the availability of clear evidence, this review provides a comprehensive summary of the current state of the evidence base. Hopefully, these findings will stimulate further research, particularly by increasing the number of contributing authors and countries to generate more robust and informative results.

The number of publications assessing anti-doping knowledge has progressively increased over the years, reflecting growing research interest in this topic. However, variations in reported knowledge levels across publication periods should be interpreted as descriptive patterns of the existing literature rather than evidence of temporal improvement or deterioration in anti-doping knowledge. In this regard, contemporary research would need to improve and standardize the tools used to measure anti-doping knowledge among different populations. Likewise, an adaptation of the design would be necessary for the future to be able to assess in a standardized way the level of training and knowledge of Athletes, ASP, and future professionals in the sports sector, and thus minimize the phenomenon of doping in sport.

In conclusion, this systematic review highlights substantial variability in reported levels of anti-doping knowledge across studies, largely driven by differences in populations, cultural settings, and measurement approaches. These factors substantially restrict the comparability and generalizability of findings. Rather than providing definitive conclusions on knowledge levels, this review underscores the urgent need for standardized, validated assessment tools and context-sensitive research designs to enable more credible comparisons and evidence-based educational strategies in the future.

## Data Availability

The original contributions presented in the study are included in the article/Supplementary Material, further inquiries can be directed to the corresponding author.
