# Supplementary material for: Exploring anti-doping knowledge level: a systematic review among athletes, students, and athlete support personnel in the sports sector
Source: Front Sports Act Living. 2026 Jun 3;8:1778209. doi: 10.3389/fspor.2026.1778209 (PMC13273595; doi:10.3389/fspor.2026.1778209)
Supplement: Supplementary file 1 [file Supplementaryfile1.docx]

Supplementary Material

# Appendix A. Full Database Search Strategies

This appendix presents the exact database-specific search strings used to retrieve records for this systematic review. These queries correspond to the conceptual search components described in the Search Strategy section of the main manuscript. All searches were executed on 20 January 2024 without post hoc modifications to the search syntax.

1. PubMed (MEDLINE)

Platform: National Library of Medicine

Search date: 20 January 2024

Search strategy:

(((doping[Title/Abstract] OR anti-doping[Title/Abstract] OR antidoping[Title/Abstract]) AND (sport*[Title/Abstract] OR 'physical activity'[Title/Abstract] OR 'physical education’[Title/Abstract])) AND (educa*[Title/Abstract] OR formati*[Title/Abstract] OR training[Title/Abstract])) AND (knowledge[Title/Abstract])

2. Scopus

Platform: Elsevier

Search date: 20 January 2024

Search strategy:

( TITLE-ABS-KEY ( doping OR anti-doping OR antidoping ) AND TITLE-ABS-KEY ( sport* OR 'physical activity' OR 'physical education' ) AND TITLE-ABS-KEY ( educa* OR formati* OR training ) AND TITLE-ABS-KEY ( knowledge ) )

3. SPORTDiscus (EBSCOhost)

Platform: EBSCOhost

Search date: 20 January 2024

Search strategy (Title):

TI ( doping OR anti-doping OR antidoping ) AND TI ( sport* OR "physical activity" OR "physical education" ) AND TI ( educa* OR formati* OR training ) AND TI knowledge

Search strategy (Abstract):

AB ( doping OR anti-doping OR antidoping ) AND AB ( sport* OR "physical activity" OR "physical education" ) AND AB ( educa* OR formati* OR training ) AND AB knowledge

4. Web of Science Core Collection

Platform: Clarivate Analytics

Indexes searched: SCI-EXPANDED, SSCI, ESCI

Search date: 20 January 2024

Search strategy:

((TS=doping) OR (TS=anti-doping) OR (TS= antidoping)) AND ((TS=sport*) OR (TS='physical activity') OR (TS='physical education')) AND ((TS=educa*) OR (TS=formati*) OR (TS=training)) AND ((TS=knowledge))
