# Supplementary material for: Exploring anti-doping knowledge level: a systematic review among athletes, students, and athlete support personnel in the sports sector
Source: Front Sports Act Living. 2026 Jun 3;8:1778209. doi: 10.3389/fspor.2026.1778209 (PMC13273595; doi:10.3389/fspor.2026.1778209)
Supplement: Supplementary file 2 [file Datasheet1.docx]

Supplementary Material

# Supplementary Figures and Tables

## Supplementary Figures


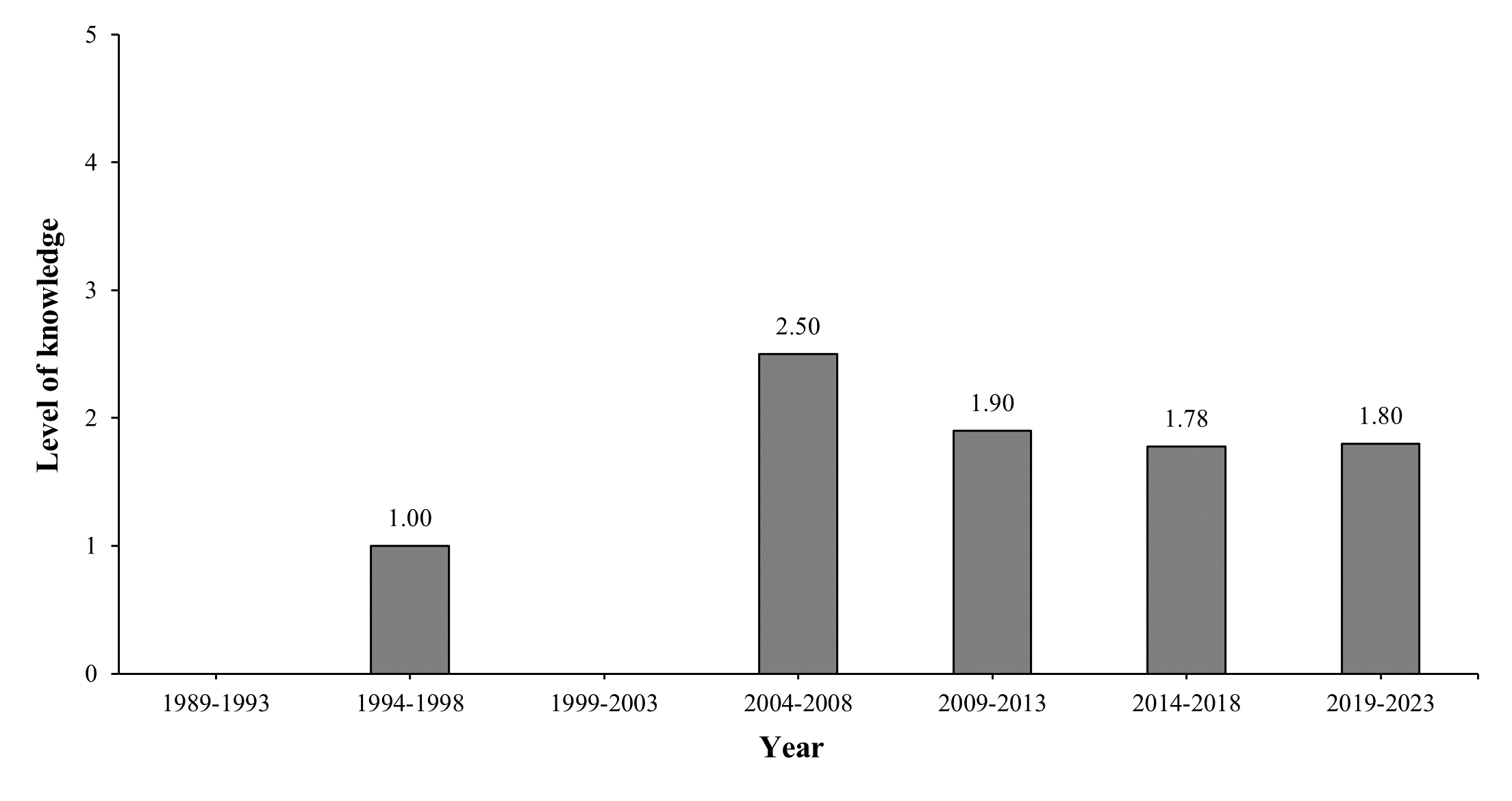


**Figure 4.** Heuristic trends in anti-doping knowledge over time, derived from descriptive and interpretive aggregation of heterogeneous study outcomes. Values represent aggregated findings from independent cross-sectional studies grouped by five-year publication periods. These data describe patterns in the published literature and should not be interpreted as longitudinal changes in anti-doping knowledge within the same populations.


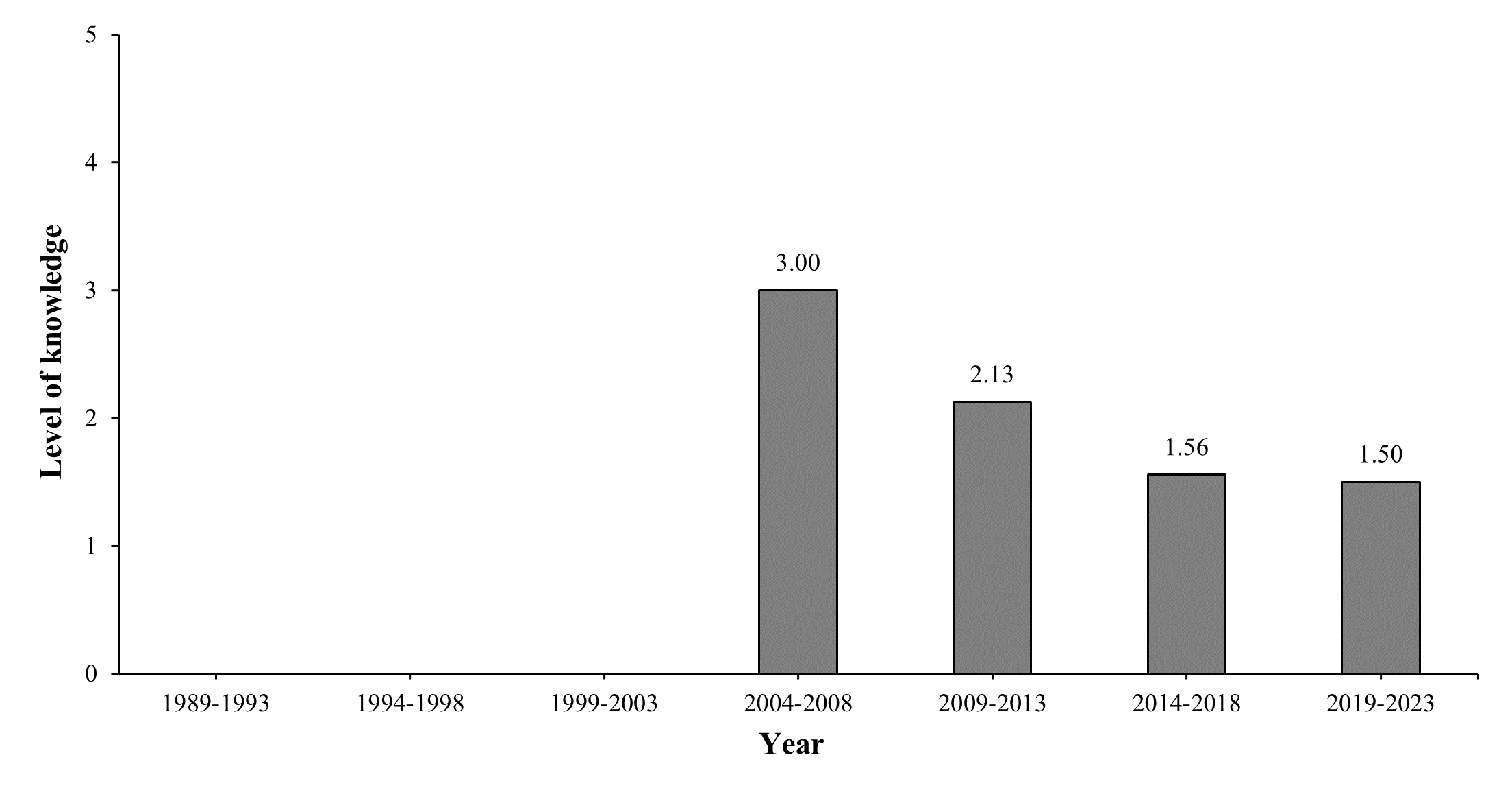


**Figure 5.** Interpretive trends in reported anti-doping knowledge among Athletes across publication periods, based on heuristic categorization of study findings. Values represent aggregated classifications derived from independent cross-sectional samples published within five-year time windows. These findings describe patterns in the published literature and should not be interpreted as longitudinal changes within the same athlete populations.


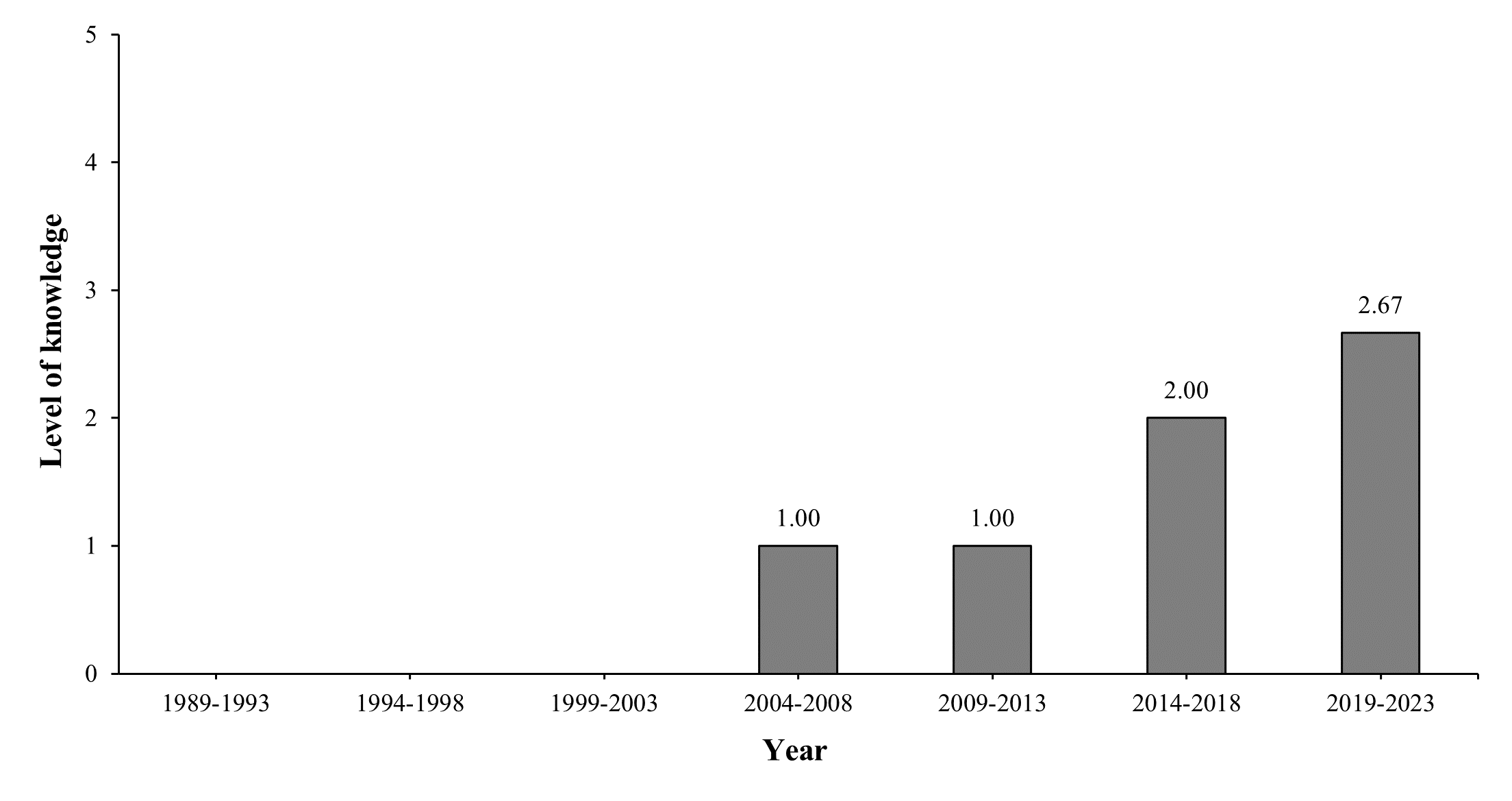


**Figure 6.** Distribution of reported anti-doping knowledge levels among Students across publication periods, based on heuristic categorization of heterogeneous study outcomes. Values represent aggregated classifications derived from independent cross-sectional samples grouped within five-year publication windows. Observed differences reflect descriptive patterns in the published literature and should not be interpreted as longitudinal changes within the same student cohorts.


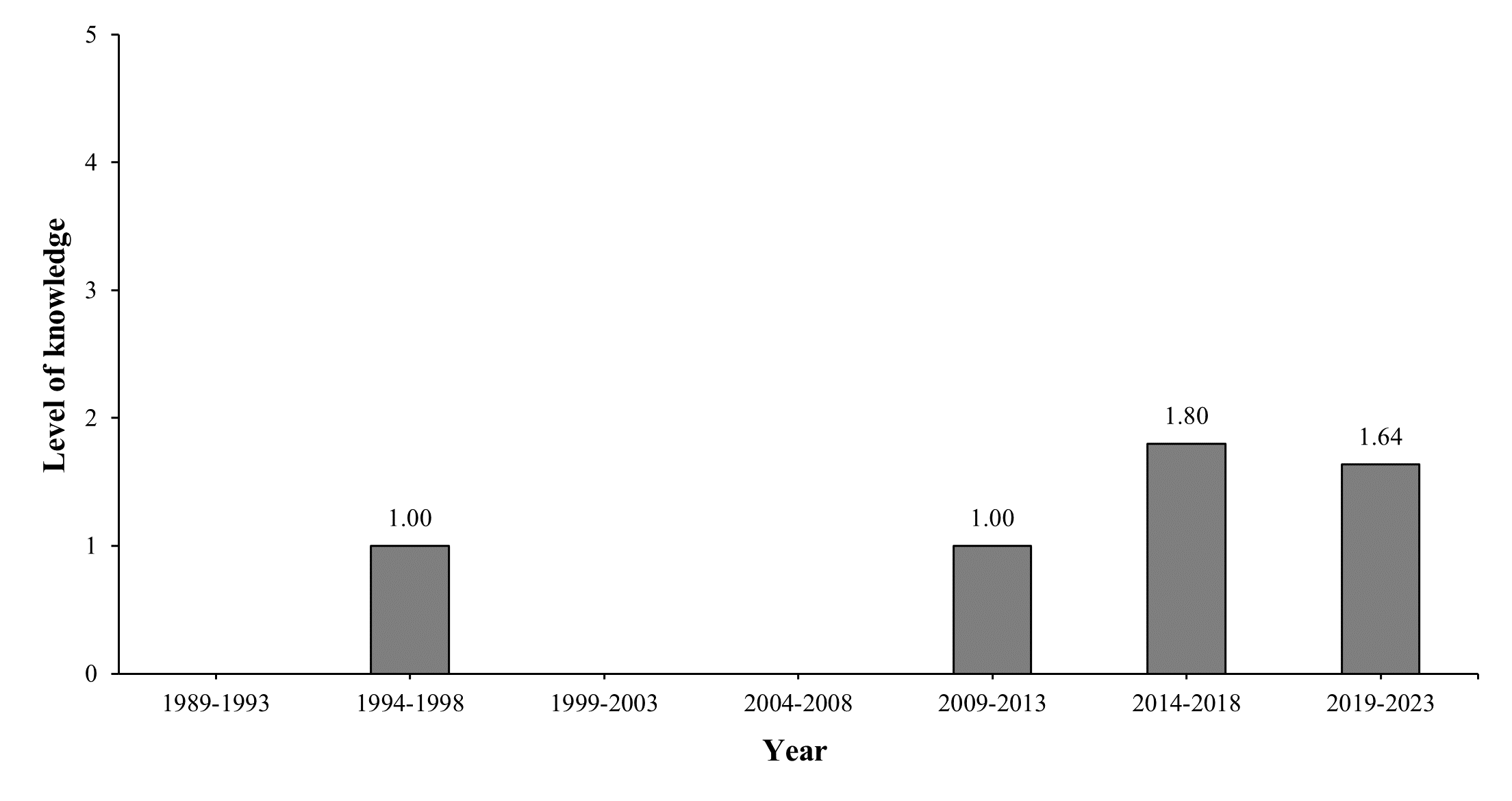


**Figure 7.** Distribution of reported anti-doping knowledge levels among Health Professional Athlete Support Personnel across publication periods, based on heuristic categorization of heterogeneous study outcomes. Values represent aggregated classifications derived from independent cross-sectional samples grouped within five-year publication windows. Findings reflect descriptive patterns in the published literature and should not be interpreted as longitudinal changes within the same populations.


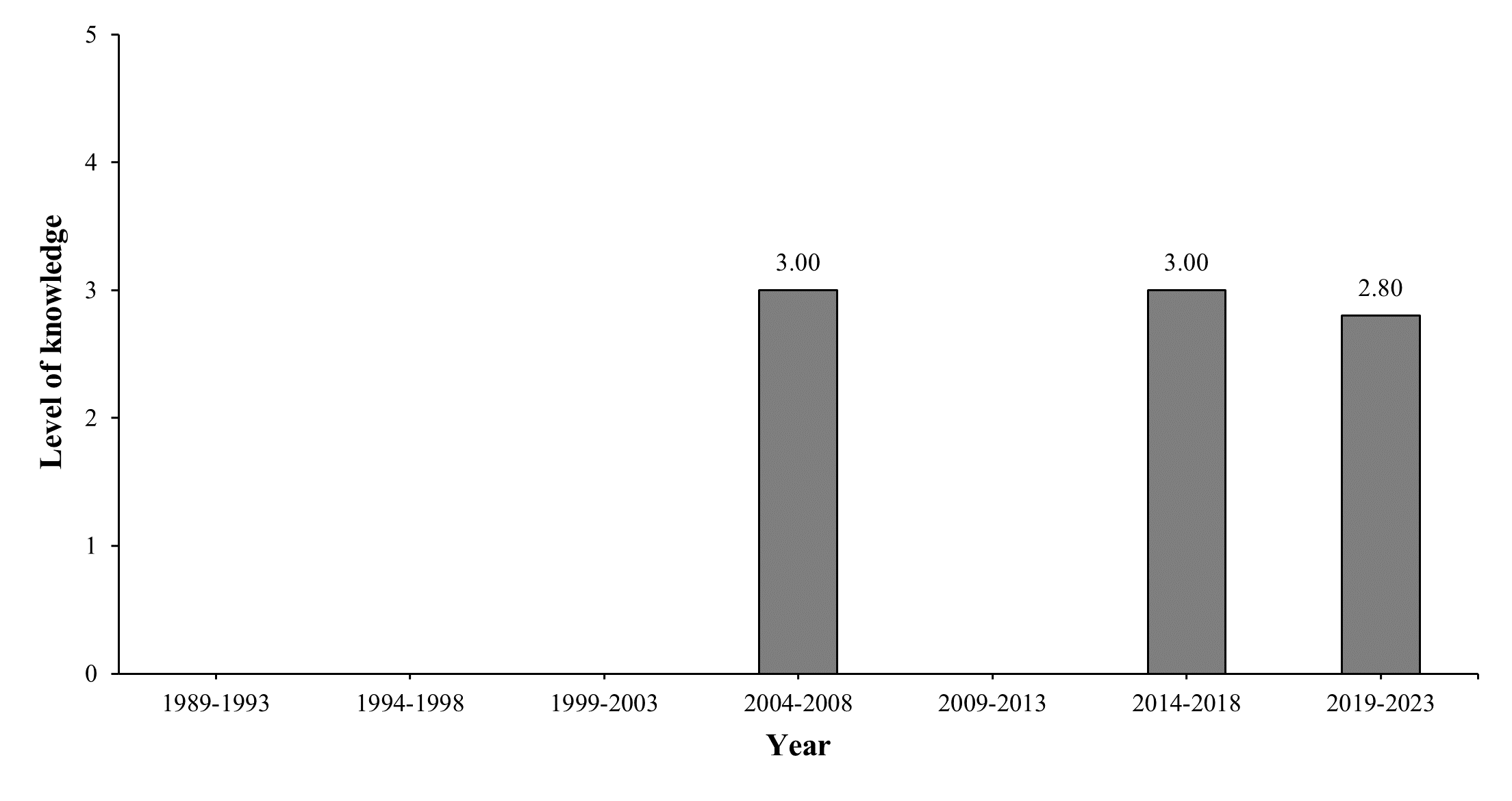


**Figure 8.** Distribution of reported anti-doping knowledge levels among Non-Health Professional Athlete Support Personnel across publication periods, based on heuristic categorization of heterogeneous study outcomes. Values represent aggregated classifications derived from independent cross-sectional samples grouped within five-year publication windows. These data reflect descriptive patterns in the published literature and should not be interpreted as longitudinal changes within the same populations over time.

## Supplementary Tables

**Table 1.** Quality assessment list of included studies and scores.

| **Studies** | **AD Knowledge Assessment** | **Descriptive**  **Data** | **AD Knowledge Level** | **Methodology Description** | **Limitations Identification** | **Instruments Quality** | **Data Collection Times** | **Total**  **Score** | **Quality**  **Level** |
| --- | --- | --- | --- | --- | --- | --- | --- | --- | --- |
| Aguilar-Navarro et al., 2022 (8) | 2 | 2 | 2 | 2 | 2 | 2 | 0 | *12 (8.571)* | T1 |
| Sajber et al., 2013 (16) | 2 | 2 | 0 | 2 | 2 | 2 | 2 | *12 (8.571)* | T1 |
| Awaisu et al., 2015 (58) | 1 | 2 | 2 | 2 | 2 | 2 | 0 | *11 (7.857)* | T1 |
| Chan et al., 2019 (57) | 1 | 2 | 2 | 2 | 2 | 2 | 0 | *11 (7.857)* | T1 |
| Jural et al., 2021 (67) | 1 | 2 | 2 | 2 | 2 | 2 | 0 | *11 (7.857)* | T1 |
| Kannan et al., 2021 (68) | 2 | 2 | 2 | 2 | 1 | 2 | 0 | *11 (7.857)* | T1 |
| Kaoche et al., 2020 (48) | 2 | 1 | 2 | 2 | 2 | 2 | 0 | *11 (7.857)* | T1 |
| Lemettilä et al., 2021 (69) | 1 | 2 | 2 | 2 | 2 | 2 | 0 | *11 (7.857)* | T1 |
| Mandic et al., 2013 (25) | 1 | 2 | 2 | 2 | 2 | 2 | 0 | *11 (7.857)* | T1 |
| Mottram et al., 2016 (64) | 1 | 2 | 2 | 2 | 2 | 2 | 0 | *11 (7.857)* | T1 |
| Aly & Taha, 2020 (70) | 1 | 2 | 1 | 2 | 2 | 2 | 0 | *10 (7.143)* | T1 |
| Bhambhani et al., 2010 (26) | 1 | 2 | 1 | 2 | 2 | 2 | 0 | *10 (7.143)* | T1 |
| Blank et al., 2014c (71) | 1 | 2 | 2 | 2 | 2 | 1 | 0 | *10 (7.143)* | T1 |
| Blank et al., 2021 (6) | 1 | 1 | 2 | 2 | 2 | 2 | 0 | *10 (7.143)* | T1 |
| Fürhapter et al., 2013 (72) | 1 | 2 | 2 | 2 | 2 | 1 | 0 | *10 (7.143)* | T1 |
| Lee et al., 2023 (73) | 0 | 2 | 2 | 2 | 2 | 2 | 0 | *10 (7.143)* | T1 |
| Morente-Sánchez et al., 2019 (74) | 1 | 2 | 1 | 2 | 2 | 2 | 0 | *10 (7.143)* | T1 |
| Orr et al., 2018 (75) | 2 | 2 | 2 | 2 | 0 | 2 | 0 | *10 (7.143)* | T1 |
| Stojanovic et al., 2019 (76) | 1 | 2 | 1 | 2 | 2 | 2 | 0 | *10 (7.143)* | T1 |
| Zhumabayeva et al., 2022 (27) | 2 | 2 | 0 | 2 | 2 | 2 | 0 | *10 (7.143)* | T1 |
| Balaravi et al., 2017 (28) | 1 | 2 | 0 | 2 | 2 | 2 | 0 | *9 (6.429)* | T1 |
| Barbadoro et al., 2018 (45) | 1 | 2 | 2 | 1 | 2 | 1 | 0 | *9 (6.429)* | T1 |
| Blank et al., 2014b (66) | 1 | 2 | 2 | 2 | 2 | 0 | 0 | *9 (6.429)* | T1 |
| Daniel, 2012 (77) | 1 | 2 | 2 | 2 | 0 | 2 | 0 | *9 (6.429)* | T1 |
| Dinçer et al., 2013 (78) | 1 | 2 | 2 | 2 | 0 | 2 | 0 | *9 (6.429)* | T1 |
| el Dine & Attia, 2022 (37) | 1 | 1 | 1 | 2 | 2 | 2 | 0 | *9 (6.429)* | T1 |
| Gebregers Hailu et al., 2021 (65) | 1 | 2 | 2 | 2 | 1 | 1 | 0 | *9 (6.429)* | T1 |
| Kondric et al., 2013 (29) | 1 | 2 | 0 | 2 | 2 | 2 | 0 | *9 (6.429)* | T1 |
| Kedir et al., 2023 (79) | 1 | 2 | 1 | 2 | 1 | 2 | 0 | *9 (6.429)* | T1 |
| Kim & Kim, 2017 (80) | 1 | 2 | 2 | 2 | 0 | 2 | 0 | *9 (6.429)* | T1 |
| Mottram et al., 2008 (21) | 2 | 2 | 2 | 2 | 0 | 1 | 0 | *9 (6.429)* | T1 |
| Murofushi et al., 2022 (50) | 1 | 2 | 2 | 2 | 1 | 1 | 0 | *9 (6.429)* | T1 |
| Nyawose et al., 2022 (81) | 1 | 1 | 1 | 2 | 2 | 2 | 0 | *9 (6.429)* | T1 |
| Rotich et al., 2023 (82) | 1 | 2 | 2 | 2 | 0 | 2 | 0 | *9 (6.429)* | T1 |
| Sas-Nowosielski & Świątkowska, 2007 (22) | 2 | 2 | 2 | 2 | 0 | 1 | 0 | *9 (6.429)* | T1 |
| Tsivitanidou et al., 2023 (83) | 1 | 2 | 2 | 2 | 1 | 1 | 0 | *9 (6.429)* | T1 |
| Álvarez Medina et al., 2019 (17) | 1 | 0 | 0 | 2 | 2 | 2 | 1 | *8 (5.714)* | T2 |
| Al Ghobain et al., 2016 (30) | 1 | 2 | 0 | 2 | 1 | 2 | 0 | *8 (5.714)* | T2 |
| Blank et al., 2014a (49) | 2 | 2 | 1 | 1 | 2 | 0 | 0 | *8 (5.714)* | T2 |
| Deng et al., 2022 (31) | 1 | 2 | 0 | 2 | 2 | 1 | 0 | *8 (5.714)* | T2 |
| El-Hammadi & Hunien, 2013 (38) | 1 | 1 | 1 | 2 | 2 | 1 | 0 | *8 (5.714)* | T2 |
| Ercan et al., 2021 (84) | 1 | 2 | 2 | 2 | 0 | 1 | 0 | *8 (5.714)* | T2 |
| Königstein et al., 2021 (85) | 1 | 1 | 2 | 2 | 0 | 2 | 0 | *8 (5.714)* | T2 |
| Loraschi et al., 2014 (86) | 1 | 2 | 2 | 1 | 2 | 0 | 0 | *8 (5.714)* | T2 |
| Morente-Sánchez & Zabala, 2015 (87) | 1 | 1 | 1 | 2 | 2 | 1 | 0 | *8 (5.714)* | T2 |
| Murofushi et al., 2018 (7) | 1 | 2 | 2 | 2 | 1 | 0 | 0 | *8 (5.714)* | T2 |
| Muwonge et al., 2015 (51) | 1 | 2 | 1 | 2 | 1 | 1 | 0 | *8 (5.714)* | T2 |
| Ozkan et al., 2020 (52) | 1 | 2 | 2 | 1 | 0 | 2 | 0 | *8 (5.714)* | T2 |
| Shibata et al., 2017 (39) | 2 | 2 | 0 | 2 | 2 | 0 | 0 | *8 (5.714)* | T2 |
| Yee et al., 2020 (88) | 1 | 0 | 2 | 2 | 1 | 2 | 0 | *8 (5.714)* | T2 |
| Allen et al., 2021 (53) | 1 | 2 | 2 | 2 | 0 | 0 | 0 | *7 (5.000)* | T2 |
| Antić & Ostojić, 2012 (54) | 0 | 2 | 2 | 1 | 2 | 0 | 0 | *7 (5.000)* | T2 |
| Azizi et al., 2012 (55) | 1 | 2 | 2 | 1 | 0 | 1 | 0 | *7 (5.000)* | T2 |
| Blank et al., 2015 (5) | 1 | 2 | 0 | 2 | 2 | 0 | 0 | *7 (5.000)* | T2 |
| Brown et al., 2013 (59) | 1 | 2 | 2 | 2 | 0 | 0 | 0 | *7 (5.000)* | T2 |
| Ćorluka et al., 2011 (89) | 1 | 2 | 2 | 2 | 0 | 0 | 0 | *7 (5.000)* | T2 |
| Engelberg et al., 2019 (90) | 1 | 2 | 2 | 2 | 0 | 0 | 0 | *7 (5.000)* | T2 |
| Engelberg & Moston, 2015 (43) | 1 | 2 | 0 | 2 | 2 | 0 | 0 | *7 (5.000)* | T2 |
| Farzin et al., 2011 (91) | 1 | 0 | 2 | 2 | 0 | 2 | 0 | *7 (5.000)* | T2 |
| Fung, 2006 (23) | 1 | 1 | 2 | 1 | 0 | 2 | 0 | *7 (5.000)* | T2 |
| Krishnan et al., 2022 (92) | 1 | 0 | 1 | 2 | 1 | 2 | 0 | *7 (5.000)* | T2 |
| Mazanov et al., 2014 (93) | 1 | 1 | 2 | 2 | 0 | 1 | 0 | *7 (5.000)* | T2 |
| Murofushi et al., 2023 (94) | 0 | 2 | 1 | 2 | 2 | 0 | 0 | *7 (5.000)* | T2 |
| Odeh et al., 2022 (40) | 0 | 2 | 1 | 2 | 0 | 2 | 0 | *7 (5.000)* | T2 |
| Pöppel & Büsch, 2019 (95) | 0 | 2 | 2 | 2 | 1 | 0 | 0 | *7 (5.000)* | T2 |
| Rintaugu & Mwangi, 2021 (60) | 1 | 2 | 2 | 2 | 0 | 0 | 0 | *7 (5.000)* | T2 |
| Seif Barghi et al., 2015 (96) | 1 | 0 | 2 | 2 | 0 | 2 | 0 | *7 (5.000)* | T2 |
| Weber et al., 2022a (32) | 1 | 2 | 0 | 2 | 2 | 0 | 0 | *7 (5.000)* | T2 |
| Weber et al., 2022b (44) | 1 | 2 | 0 | 2 | 2 | 0 | 0 | *7 (5.000)* | T2 |
| Yalçın et al., 2019 (97) | 1 | 1 | 2 | 1 | 0 | 2 | 0 | *7 (5.000)* | T2 |
| Zia & Javed, 2020 (63) | 1 | 2 | 2 | 1 | 1 | 0 | 0 | *7 (5.000)* | T2 |
| Zmuda et al., 2023 (98) | 1 | 1 | 2 | 2 | 1 | 0 | 0 | *7 (5.000)* | T2 |
| Ceylan et al., 2020 (33) | 1 | 1 | 0 | 2 | 0 | 2 | 0 | *6 (4.286)* | T3 |
| Greenbaum et al., 2023 (99) | 1 | 2 | 1 | 2 | 0 | 0 | 0 | *6 (4.286)* | T3 |
| Nolte et al., 2014 (100) | 1 | 2 | 1 | 1 | 0 | 1 | 0 | *6 (4.286)* | T3 |
| Scarpino et al., 1990 (34) | 1 | 2 | 0 | 2 | 0 | 1 | 0 | *6 (4.286)* | T3 |
| Sepriani et al., 2022 (101) | 2 | 1 | 2 | 1 | 0 | 0 | 0 | *6 (4.286)* | T3 |
| Sertbaş et al., 2015 (102) | 1 | 2 | 1 | 0 | 0 | 2 | 0 | *6 (4.286)* | T3 |
| Uslu et al., 2020 (103) | 1 | 2 | 2 | 1 | 0 | 0 | 0 | *6 (4.286)* | T3 |
| Wanjek et al., 2007 (24) | 0 | 2 | 2 | 2 | 0 | 0 | 0 | *6 (4.286)* | T3 |
| Yildiz, 2019 (104) | 1 | 2 | 2 | 1 | 0 | 0 | 0 | *6 (4.286)* | T3 |
| Antić, 2017 (105) | 1 | 1 | 2 | 1 | 0 | 0 | 0 | *5 (3.571)* | T3 |
| Duda & Stula, 2022 (106) | 1 | 0 | 2 | 0 | 0 | 2 | 0 | *5 (3.571)* | T3 |
| Fagnani et al., 2018 (18) | 1 | 1 | 0 | 2 | 0 | 0 | 1 | *5 (3.571)* | T3 |
| Poussel et al., 2013 (19) | 1 | 1 | 0 | 2 | 0 | 0 | 1 | *5 (3.571)* | T3 |
| Roman et al., 2022 (41) | 1 | 0 | 0 | 2 | 2 | 0 | 0 | *5 (3.571)* | T3 |
| Starzak et al., 2016 (107) | 1 | 1 | 2 | 0 | 1 | 0 | 0 | *5 (3.571)* | T3 |
| Greenway & Greenway, 1997 (20) | 2 | 0 | 2 | 0 | 0 | 0 | 0 | *4 (2.857)* | T3 |
| Salih & Younis, 2021 (42) | 1 | 1 | 0 | 1 | 1 | 0 | 0 | *4 (2.857)* | T3 |
| Woods & Moynihan, 2009 (108) | 1 | 1 | 2 | 0 | 0 | 0 | 0 | *4 (2.857)* | T3 |
| Massucci et al., 2019 (35) | 1 | 0 | 0 | 2 | 0 | 0 | 0 | *3 (2.143)* | T3 |
| Pavlović & Idrizović, 2013 (109) | 1 | 0 | 2 | 0 | 0 | 0 | 0 | *3 (2.143)* | T3 |
| Laure & Reisenberger, 1995 (36) | 0 | 0 | 0 | 2 | 0 | 0 | 0 | *2 (1.429)* | T3 |
|  |  |  |  |  |  |  |  |  |  |

Parameter 1: Does the evaluation of anti-doping knowledge clearly separate from other types of evaluations (attitude toward doping, doping practices, experience) in its objective(s)? 0: No; 1: Yes (not clearly); 2: Yes

Parameter 2: Does it identify the descriptive data of the participants? 0: Less than 3; 1: Between 3 and 5 (inclusive); 2: More than 5.

Parameter 3: Does it clearly identify the level of anti-doping knowledge? 0: No; 1: Yes (imprecise or doubtful); 2: Yes.

Parameter 4: Is the methodological process applied clearly and precisely described? 0: Basic; 1: Intermediate; 2: Complete.

Parameter 5: Are the limitations of the study described? 0: No; 1: Yes (incompletely); 2: Yes.

Parameter 6: Are validated questionnaires used (Quantitative studies)? 0: Ad-Hoc; 1: Pre-tested/Pilot study; 2: Validated. How many data sources are used (Qualitative and Mixed methods studies): 0: 1 data source; 1: 2 data sources; 2: 3 data sources or more.

Parameter 7: How many data collection moments are conducted during the study? 0: Cross-sectional study (at a single point in time); 1: Longitudinal study (at least 2 over a specific period); 2: Longitudinal study (3 or more over a specific period).

T1: Tertile 1; T2: Tertile 2; T3: Tertile 3.

Table 2. Countries with the highest number of publications on anti-doping knowledge assessment.

| Number of studies | Percentage of studies (%) | Countries (number of countries) |
| --- | --- | --- |
| 8 | 8.60% | Austria; Turkey (2) |
| 6 | 6.45% | Australia (1) |
| 4 | 4.30% | Spain; Italy; Japan; United Kingdom (4) |
| 3 | 3.23% | Iran; Poland; Serbia; South Africa (4) |
| 2 | 2.15% | Germany; Bosnia & Herzegovina; Egypt; Ethiopia; France; India; Kenya; Malaysia; Slovenia; Switzerland (10) |
| 1 | 1.08% | Brazil; Canada; China; Cyprus; Croatia; United States; Finland; Ghana; Hong Kong; Indonesia; Iraq; Ireland; Jordan; Kazakhstan; Malawi; Nigeria; Pakistan; Qatar; Republic of Korea; Saudi Arabia; Syria; Taiwan; Uganda (23) |

**Table 3.** Athletes anti-doping knowledge level.

| Level of knowledge | Keywords | Statements | Study | Design & Methods |
| --- | --- | --- | --- | --- |
| · Low | Deficit (1)  Inadequate (2)  Insufficient (5)  Lack of knowledge (5)  Low (3)  Poor (6) | Athletes have *insufficient* knowledge about different aspects of doping  The knowledge of tertiary student sportspersons on doping substances is *poor*  There is *insufficient* knowledge about doping in football  The knowledge about the support and the problem of doping in sport is *insufficient*  Wrestlers have *poor* knowledge in doping  *Insufficient* knowledge of PET towards doping  There is a *low* level of doping knowledge among athletes  Athlete awareness regarding anti-doping agencies and ADRVs was *poor*  Doping knowledge was *poor* and biased  The existence of an important *lack of knowledge* about doping has been demonstrated  University athletes had a *low* rate of correct answers on the ALPHA test  Anti-doping knowledge measured by the level of willingness to learn was *inadequate*  There is an existing knowledge *deficit* prevalent among university athletes  *Lack of knowledge* in certain aspects of doping observed among athletes  There is still a *lack of knowledge*  Substantial *lack of knowledge* of the WADA List of Prohibited substances  Elite athletes have *poor* knowledge about doping  Football players have *poor* knowledge about doping  High-school athletes *lack* *basic* *knowledge* about doping  Volleyball players had *inadequate* information about doping knowledge and gene doping  Athletes did *not* *have* *sufficient* knowledge level about doping  Bodybuilding athletes had *low* levels of knowledge concerning doping | Antić & Ostojić, 2012 (54)  Brown et al., 2013 (59)  Ćorluka et al., 2011 (89)  Duda & Stula, 2022 (106)  Farzin et al., 2011 (91)  Kannan et al., 2021 (68)  Kim & Kim, 2017 (80)  Krishnan et al., 2022 (92)  Loraschi et al., 2014 (86)  Morente-Sánchez et al., 2019 (74)  Murofushi et al., 2018 (7)  Murofushi et al., 2022 (50)  Murofushi et al., 2023 (94)  Muwonge et al., 2015 (51)  Nolte et al., 2014 (100)  Nyawose et al., 2022 (81)  Ozkan et al., 2020 (52)  Seif Barghi et al., 2015 (96)  Tsivitanidou et al., 2023 (83)  Uslu et al., 2020 (103)  Yalçın et al., 2019 (97)  Yildiz, 2019 (104) | CS, Q  CS, Q  CS, Q  CS, Q  CS, Q  CS, Q  CS, Q  CS, Q  CS, Q  CS, Q  CS, Q  CS, Q  CS, Q  CS, Q  CS, Q  CS, Q  CS, Q  CS, Q  CS, Q  CS, Q  CS, Q  CS, I |
| · Limited | Limited (4)  Not enough (2) | *Limited* knowledge and understanding of the WADA Prohibited List and TUE policy concerning asthma medication  The athletes did *not have enough* knowledge about doping  Respondents had a *limited* knowledge of OTC medication in reference to anti-doping  Athletes showed a *limited* understanding of the Prohibited Substances List  Athletes during the PON XX Papua 2021 have *limited* knowledge of doping  Athletes who have sports education do *not* *have* *enough* knowledge about doping | Allen et al., 2021 (53)  Dinçer et al., 2013 (78)  Mottram et al., 2008 (21)  Orr et al., 2018 (75)  Sepriani et al., 2022 (101)  Sertbaş et al., 2015 (102) | CS, I  CS, Q  CS, Q  CS, Q  CS, Q  CS, Q |
| · Basic | Fair (1) | *Fair* level of knowledge on the harmful effects and health implications of drug abuse for performance enhancement among study participants | Daniel, 2012 (77) | CS, Q |
| · Good | Adequate (1)  Good (2)  Moderate (4) | The knowledge of rowers about supplements and doping was *moderate*  This study generally shows a *good* knowledge about doping substance and methods  Knowledge about legal consequences was *moderate*  Results show a *good* overall state of knowledge of athletes regarding doping in sport  Football athletes (77%) had *adequate* knowledge of doping  Actual knowledge was *moderate*-to-good  The general index of correct answers indicates a *moderate* level of knowledge | Azizi et al., 2012 (55)  Blank et al., 2014a (49)  Blank et al., 2021 (6)  Fürhapter et al., 2013 (28)  Kaoche et al., 2020 (48)  Königstein et al., 2021 (85)  Sas-Nowosielski y Świątkowska, 2007 (22) | CS, Q  CS, Q  CS, Q  CS, Q  CS, Q  CS, Q  CS, Q |
| CS: Cross-Sectional Design; Q: Questionnaire; I: Interview | | | | |

**Table 4.** Students anti-doping knowledge level.

| Level of knowledge | Keywords | Statements | Study | Design & Methods |
| --- | --- | --- | --- | --- |
| · Low | Insufficient (2)  Lack of knowledge (1)  Poor (3) | Most students showed *lack of knowledge*  Students had *poor* knowledge about doping  The knowledge of tertiary student sportspersons on doping substances is *poor*  Students knowledge about the use of doping and its negative effects are *insufficient*  The level of knowledge of personal trainer students on doping is still *insufficient*  Specific knowledge of doping revealed a *poor* state of awareness in general | Aly & Taha, 2020 (70)  Barbadoro et al., 2018 (45)  Brown et al., 2013 (59)  Pavlović & Idrizović, 2013 (109)  Zmuda et al., 2023 (98)  Wanjek et al., 2007 (24) | CS, Q  CS, Q  CS, Q  CS, Q  CS, Q  CS, Q |
| · Limited | Not enough (2) | Students who have sports education do *not* *have* *enough* knowledge about doping  The student population is *not* *knowledgeable* *enough* about the problem of prohibited substance use and the negative effects the substances have on one’s health | Sertbaş et al., 2015 (102)  Stojanovic et al., 2019 (76) | CS, Q  CS, Q |
| · Basic | Basic (1) | Pharmacy students had some *basic* knowledge of anti-doping in sports | Awaisu et al., 2015 (58) | CS, Q |
| · Good | Moderate (2)  Suboptimal (1) | The level of anti-doping knowledge of sports sciences students was *suboptimal*  Pharmacy students had a *moderate* level of knowledge  University students have *moderate* knowledge of doping and substances that enhance sports performance | Aguilar-Navarro et al., 2022 (8)  Chan et al., 2019 (57)  Rintaugu & Mwangi, 2021 (60) | CS, Q  CS, Q  CS, Q |
| CS: Cross-Sectional Design; Q: Questionnaire | | | | |

**Table 5.** Health Professionals ASP anti-doping knowledge level.

| Level of knowledge | Keywords | Statements | Study | Design & Methods |
| --- | --- | --- | --- | --- |
| · Low | Inadequate (1)  Insufficient (3)  Lack of knowledge (3)  Low (2)  Poor (4) | General practitioners have *insufficient* knowledge of different aspects of doping in sports  A *lack of knowledge* among sports physicians was found about key issues in doping  The knowledge level of intern physicians on nutritional ergogenic supplements and doping was found to be *insufficient*  Pharmacists have a *low* knowledge score  *Lack of* *core* *knowledge* and resources to deliver comprehensive care to prevent harm and protect athlete-patients from anti-doping violations  General practitioners' knowledge of which substances are prohibited in sports is *poor*  Dentists have *insufficient* knowledge about sports doping  Most pharmacy professionals *lack* *doping-specific* *knowledge*  Pharmacists reported their knowledge of anti-doping counselling to be *poor*  General Practitioners have *inadequate* doping knowledge  Both Ps and GPs admitted a *poor* knowledge of doping-related matters  GPs have *poor* knowledge of doping in sport  Physiotherapists had a *low* knowledge of doping | Antić, 2017 (105)  Blank et al., 2014b (66)  Ercan et al., 2021 (84)  Gebregers Hailu et al., 2021 (65)  Greenbaum et al., 2023 (99)  Greenway & Greenway, 1997 (20  Jural et al., 2021 (67)  Kedir et al., 2023 (79)  Lemettilä et al., 2021 (69)  Rotich et al., 2023 (82)  Starzak et al., 2016 (107)  Woods & Moynihan, 2009 (108)  Zia & Javed, 2020 (63) | CS, Q  CS, Q  CS, Q  CS, Q  CS, TE  CS, Q  CS, Q  CS, Q  CS, Q  CS, Q  CS, Q  CS, Q  CS, Q |
| · Limited | Limited (1) | *Limited* pharmacists’ knowledge in providing advice to athletes about medication use | Yee et al., 2020 (88) | CS, Q |
| · Basic | Basic (1)  Reasonable (1) | ASP has a *reasonable* grasp of the overall anti-doping system as it applies to athletes  Pharmacists demonstrated a *basic* level of knowledge of doping and anti-doping | Mazanov et al., 2014 (93)  Mottram et al., 2016 (64) | CS, Q, I  CS, Q |
| · Good | Moderate (2) | Knowledge about legal consequences was *moderate*  Respondents reported a *moderate* anti-doping knowledge | Blank et al., 2021 (6)  Lee et al., 2023 (73) | CS, Q  CS, Q |
| CS: Cross-Sectional Design; Q: Questionnaire; I: Interview; TE: Telephone Enquiry | | | | |

**Table 6.** Non-Health Professionals ASP anti-doping knowledge level.

| Level of knowledge | Keywords | Statements | Study | Design & Methods |
| --- | --- | --- | --- | --- |
| · Low | Insufficient (1)  Lack of knowledge (1)  Poor (2) | Knowledge of anti-doping control systems was very *poor*  *Insufficient* knowledge of PET towards doping  *Lack of knowledge* about doping  Coaches have *poor* knowledge about doping | Engelberg et al., 2019 (90)  Kannan et al., 2021 (68)  Morente-Sánchez & Zabala, 2015 (87)  Seif Barghi et al., 2015 (96) | CS, I  CS, Q  CS, Q  CS, Q |
| · Limited | - | - | - | - |
| · Basic | Reasonable (1)  Fair (1) | The present respondents’ actual knowledge on PEDs was *fair*  ASP have a *reasonable* grasp of the overall anti-doping system as it applies to athletes | Fung, 2006 (23)  Mazanov et al., 2014 (93) | CS, Q  CS, Q, I |
| · Good | Adequate (1)  Good (1)  Moderate (1)  Satisfactory (1) | Coaches generally show a *good* level of actual knowledge about doping substances, methods and side effects  Knowledge about legal consequences was *moderate*  Coaches (45.8%) and sponsors (60%) had *adequate* knowledge of doping  Coaches indicate a *satisfying* knowledge concerning doping substances | Blank et al., 2014c (71)  Blank et al., 2021 (6)  Kaoche et al., 2020 (48)  Pöppel & Büsch, 2019 (95) | CS, Q  CS, Q  CS, Q  CS, Q |
| CS: Cross-Sectional Design; Q: Questionnaire; I: Interview | | | | |

## References

# Ministers CoECo. Resolution (67) 12 on the Doping of Athletes (Adopted by the Ministers’ on 29th June 1967). Strasbourg: Council of Europe (1967).

# Backhouse SH. A behaviourally informed approach to reducing the risk of inadvertent anti-doping rule violations from supplement use. Sports Med. (2023) 53(S1):67–84. [doi: 10.1007/s40279-023-01933-x](https://www.doi.org/10.1007/s40279-023-01933-x)

# WADA. World Anti-Doping Code 2021: World Anti-Doping Agency. (2021). Available online at: <https://www.wada-ama.org/en/resources/world-anti-doping-code-and-international-standards/world-anti-doping-code>

# Morente-Sánchez J, Zabala M. Doping in sport: a review of elite Athletes’ attitudes, beliefs, and knowledge. Sports Med. (2013) 43(6):395–411. [doi: 10.1007/s40279-013-0037-x](https://doi.org/10.1007/s40279-013-0037-x)

# Blank C, Leichtfried V, Schaiter R, Fürhapter C, Müller D, Schobersberger W. Doping in sports: knowledge and attitudes among parents of Austrian junior athletes. Scand J Med Sci Sports. (2015) 25(1):116–24. [doi: 10.1111/sms.12168](https://doi.org/10.1111/sms.12168)

# Blank C, Flatscher-Thöni M, Gatterer K, Happ E, Schobersberger W, Stühlinger V. Doping sanctions in sport: knowledge and perception of (legal) consequences of doping—an explorative study in Austria. J Risk Financ Manag. (2021) 14(12):603. [doi: 10.3390/jrfm14120603](https://doi.org/10.3390/jrfm14120603)

# Murofushi Y, Kawata Y, Kamimura A, Hirosawa M, Shibata N. Impact of anti-doping education and doping control experience on anti-doping knowledge in Japanese university athletes: a cross-sectional study. Subst Abuse Treat Prev Policy. (2018) 13:44. [doi: 10.1186/s13011-018-0178-x](https://doi.org/10.1186/s13011-018-0178-x)

# Aguilar-Navarro M, Salas-Montoro JA, Pino-Ortega J, Salinero JJ, González Mohíno F, Alcaraz-Rodríguez V, et al. Anti-doping knowledge of students undertaking Bachelor’s degrees in sports sciences in Spain. Nutrients. (2022) 14(21):4523. [doi: 10.3390/nu14214523](https://doi.org/10.3390/nu14214523)

# WADA. International Standard for Education: World Anti-Doping Agency. (2021). Available online at: <https://www.wada-ama.org/en/resources/world-antidoping-code-and-international-standards/international-standard-education-ise>

# García-Grimau E, De La Vega R, Casado A. Moral disengagement, social norms, and motivational profiles influence attitudes toward doping among Spanish athletics coaches. Front Sports Act Living. (2022) 4:842959. [doi: 10.3389/fspor.2022.842959](https://doi.org/10.3389/fspor.2022.842959)

# Backhouse SH, McKenna J. Reviewing Coaches’ knowledge, attitudes and beliefs regarding doping in sport. Int J Sports Sci Coach. (2012) 7(1):167–75. [doi: 10.1260/ 1747-9541.7.1.167](https://doi.org/10.1260/1747-9541.7.1.167)

# Page MJ, Moher D, Bossuyt PM, Boutron I, Hoffmann TC, Mulrow CD, et al. PRISMA 2020 Explanation and elaboration: updated guidance and exemplars for reporting systematic reviews. Br Med J. (2021) 372:n160. [doi: 10.1136/bmj.n160](https://doi.org/10.1136/bmj.n160)

# Moher D, Schulz KF, Altman DG. The CONSORT statement: revised recommendations for improving the quality of reports of parallel group randomized trials. BMC Med Res Methodol. (2001) 1(1). [doi: 10.1186/1471-2288-1-2](https://doi.org/10.1186/1471-2288-1-2)

# Vandenbroucke JP, Elm V, Altman E, Gøtzsche DG, Mulrow PC, Pocock CD, et al. Strengthening the reporting of observational studies in epidemiology (STROBE). Epidemiology. (2007) 18(6):805–35. [doi: 10.1097/ede.0b013e3181577511](https://doi.org/10.1097/EDE.0b013e3181577511)

# Pozo P, Grao-Cruces A, Pérez-Ordás R. Teaching personal and social responsibility model-based programmes in physical education. Eur Phy Educ Rev. (2018) 24(1):56–75. [doi: 10.1177/1356336X16664749](https://doi.org/10.1177/1356336X16664749)

# Sajber D, Rodek J, Escalante Y, Olujic D, Sekulic D. Sport nutrition and doping factors in swimming; parallel analysis among athletes and coaches. Coll Antropol. (2013) 37:179–86.

# Medina JA, Marqueta PM, Grao-Cruces A, Blanco EO, Lorente VM, Nuviala AN. Effectiveness of a school-based doping prevention programme in Spanish adolescents. J Hum Sport Exerc. (2019) 14(4):813–20. [doi: 10.14198/JHSE.2019.144.10](https://doi.org/10.14198/jhse.2019.144.10)

# Fagnani F, Faiola F, Tomassi G, Di Cagno A, Pigozzi F, Borrione N. Dissemination of the “WADA’s outreach model on antidoping” in youth sport. Med Dello Sport. (2018) 71(3):474–89. [doi: 10.23736/S0025-7826.18.03397-5](https://doi.org/10.23736/S0025-7826.18.03397-5)

# Poussel M, Laure P, Latarche C, Laroppe J, Schwitzer M, Koch JP, et al. Specific teaching about doping in sport helps medical students to meet prevention needs. Sci Sports. (2013) 28(5):274–80. [doi: 10.1016/j.scispo.2013.01.009](https://doi.org/10.1016/j.scispo.2013.01.009)

# Greenway P, Greenway M. General practitioner knowledge of prohibited substances in sport. Br J Sports Med. (1997) 31(2):129–31. [doi: 10.1136/bjsm.31.2.129](https://doi.org/10.1136/bjsm.31.2.129)

# Mottram D, Chester N, Atkinson C, Goode D. Athletes’ knowledge and views on OTC medication. Int J Sports Med. (2008) 29(10):851–5. [doi: 10.1055/s-2008-1038403](https://doi.org/10.1055/s-2008-1038403)

# Sas-Nowosielski K, Świątkowska L. The knowledge of the world anti-doping code among Polish athletes and their attitudes toward doping and anti-doping policy. Hum Mov. (2007) 8(1):57–64.

# Fung L. Performance enhancement drugs: knowledge, attitude, and intended behavior among community coaches in Hong Kong. Sport J. (2006).

# Wanjek B, Rosendahl J, Strauss B, Gabriel HH. Doping, drugs and drug abuse among adolescents in the State of Thuringia (Germany): prevalence, knowledge and attitudes. Int J Sports Med. (2007) 28(4):346–53. [doi: 10.1055/s-2006-924353](https://doi.org/10.1055/s-2006-924353)

# Mandic GF, Peric M, Krzelj L, Stankovic S, Zenic N. Sports nutrition and doping factors in synchronized swimming: parallel analysis among athletes and coaches. J Sports Sci Med. (2013) 12(4):753–60.

# Bhambhani Y, Mactavish J, Warren S, Thompson WR, Webborn A, Bressan E, et al. Boosting in athletes with high-level spinal cord injury: knowledge, incidence and attitudes of athletes in paralympic sport. Disabil Rehabil. (2010) 32(26):2172–90. [doi: 10.3109/09638288.2010.505678](https://doi.org/10.3109/09638288.2010.505678)

# Zhumabayeva G, Kapanova G, Vinnikov D, Bakasheva M, Abdulla V, Grjibovski A. Knowledge and experience of Kazakhstan athletes in anti-doping and the impact of past educational intervention. Subst Abuse Treat Prev Policy. (2022) 17(1):32. [doi: 10.1186/s13011-022-00461-7](https://doi.org/10.1186/s13011-022-00461-7)

# Balaravi B, Qi C, Jin C, Lw S, Ramadas A, Karppaya H. Knowledge and attitude related to nutritional supplements and risk of doping among national elite athletes in Malaysia. Malays J Nutr. (2017) 23.

# Kondric M, Sekulic D, Uljevic O, Gabrilo G, Zvan M. Sport nutrition and doping in tennis: an analysis of Athletes’ attitudes and knowledge. J Sports Sci Med. (2013) 12(2):290–7.

# Al Ghobain M, Konbaz MS, Almassad A, Alsultan A, Al Shubaili M, AlShabanh O. Prevalence, knowledge and attitude of prohibited substances use (doping) among Saudi sport players. Subst Abuse Treat Prev Policy. (2016) 11:14. [doi: 10.1186/s13011-016-0058-1](https://doi.org/10.1186/s13011-016-0058-1)

# Deng Z, Guo J, Wang D, Huang T, Chen Z. Effectiveness of the world anti-doping agency’s e-learning programme for anti-doping education on knowledge of, explicit and implicit attitudes towards, and likelihood of doping among Chinese college athletes and non-athletes. Subst Abuse Treat Prev Policy. (2022) 17(1):31. [doi: 10.1186/s13011-022-00459-1](https://doi.org/10.1186/s13011-022-00459-1)

# Weber K, Patterson LB, Blank C. An exploration of doping-related perceptions and knowledge of disabled elite athletes in the UK and Austria. Psychol Sport Exerc. (2022) 58:102061. [doi: 10.1016/j.psychsport.2021.102061](https://doi.org/10.1016/j.psychsport.2021.102061)

# Ceylan A, Calik F, Geri S, Sen M, Ece C, Sumer M. Determining the knowledge levels and opinions of athletes who is age under-16 in different branches about doping and ergogenic aids. Prog Nutr. (2020) 22(4). [doi: 10.23751/pn.v22i4.10984](https://doi.org/10.23751/pn.v22i4.10984)

# Scarpino V, Arrigo A, Benzi G, Garattini S, La Vecchia C, Bernardi LR, et al. Evaluation of prevalence of ‘doping’ among Italian athletes. Lancet. (1990) 336:1048–50. [doi: 10.1016/0140-6736(90)92502-9](https://doi.org/10.1016/0140-6736(90)92502-9)

# Masucci MA, Butryn TM, Johnson JA. Knowledge and perceptions of doping practices and anti-doping education among elite North American female triathletes. Perform Enhanc Health. (2019) 6(3):121–8. [doi: 10.1016/j.peh.2019.02.001](https://doi.org/10.1016/j.peh.2019.02.001)

# Laure P, Reinsberger H. Doping and high-level endurance walkers. Knowledge and representation of a prohibited practice. J Sports Med Phys Fitness. (1995) 35(3):228–31.

# el Dine F, Attia MH. Assessment of knowledge, perception, attitude, and use of performance-enhancing substances among students of faculty of medicine, alexandria university, Egypt: a pilot study. Egypt J Forensic Sci. (2022) 12(1):34. [doi: 10.1186/s41935-022-00290-6](https://doi.org/10.1186/s41935-022-00290-6)

# El-Hammadi M, Hunien B. Exploring knowledge, attitudes and abuse concerning doping in sport among Syrian pharmacy students. Pharmacy. (2013) 1:94–106. [doi: 10.3390/pharmacy1020094](https://doi.org/10.3390/pharmacy1020094)

# Shibata K, Ichikawa K, Kurata N. Knowledge of pharmacy students about doping, and the need for doping education: a questionnaire survey. BMC Res Notes. (2017) 10(1):396. [doi: 10.1186/s13104-017-2713-7](https://doi.org/10.1186/s13104-017-2713-7)

# Odeh M, Tailakh HM, Al Bawab AQF, Elsahoryi NA, Alzoubi KH. A comprehensive assessment of knowledge, attitudes, and practicalities related to doping agents use among Jordanians. Clin Pract Epidemiol Ment Health. (2022) 18(1):e174501792202280. [doi: 10.2174/17450179-v18-e2202280](http://dx.doi.org/10.2174/17450179-v18-e2202280)

# Roman G, Phillippe T, Ernst K, German C. Doping and the sports Physician’s role —the Swiss experience. SEMS J. (2022) 70(3):8–12. [doi: 10.34045/SEMS/2022/15](https://doi.org/10.34045/SEMS/2022/15)

# Salih M, Younis A. Knowledge, attitude, and behaviour regarding doping in sports among physicians and pharmacists: a questionnaire-based study. J Adv Pharm Educ Res. (2021) 11:29–35. [doi: 10.51847/TIT76VnUlP](https://doi.org/10.51847/TIT76VnUlP)

# Engelberg T, Moston S. Inside the locker room: a qualitative study of coaches’ anti-doping knowledge, beliefs and attitudes. Sport Soc. (2015). [doi: 10.1080/17430437.2015.1096244](https://doi.org/10.1080/17430437.2015.1096244)

# Weber K, Patterson LB, Blank C. Doping in disabled elite sport: perceptions, knowledge and opinions from the of German and UK coaches. Psychol Sport Exerc. (2022) 62:102233. [doi: 10.1016/j.psychsport.2022.102233](https://doi.org/10.1016/j.psychsport.2022.102233)

# Barbadoro P, Ponzio E, Marigliano A, Vincitorio D, Tondo D, Scandali E, et al. Study of knowledge, attitude, and consumption in Italian students towards the intake of performance-enhancing substances and energy-food supplements. Med Dello Sport. (2018) 71(4):658–66. [doi: 10.23736/S0025-7826.18.03406-3](https://doi.org/10.23736/S0025-7826.18.03406-3)

# WADA. International Standard for Testing and Investigations: World AntiDoping Agency. (2021). Available online at: <https://www.wada-ama.org/en/resources/world-anti-doping-code-and-international-standards/internationalstandard-testing-and>

# Heaney S, O’Connor H, Michael S, Gifford J, Naughton G. Nutrition knowledge in athletes: a systematic review. Int J Sport Nutr Exerc Metab. (2011) 21(3):248–61. [doi: 10.1123/ijsnem.21.3.248](https://doi.org/10.1123/ijsnem.21.3.248)

# Kaoche JMC, Rintaugu EG, Kamenju JW, Mwangi FM. Knowledge on doping among football athletes, coaches and sponsors in Malawi. (2020) 11:5–13. [doi: 10.5897/JPESM2020.0346](https://doi.org/10.5897/JPESM2020.0346)

# Blank C, Leichtfried V, Schaiter R, Müller D, Schobersberger W. Associations between doping knowledge, -susceptibility and substance use of Austrian junior elite athletes? Jacobs J Sports Med. (2014) 1:1–8.

# Murofushi Y, Kawata Y, Yamaguchi S, Nakamura M, Takazawa Y, Naito H. Relationship between the level of willingness to learn about anti-doping and objective knowledge among Japanese university athletes: a cross-sectional study. Front Sports Act Living. (2022) 4:955636. [doi: 10.3389/fspor.2022.955636](https://doi.org/10.3389/fspor.2022.955636)

# Muwonge H, Zavuga R, Kabenge PA. Doping knowledge, attitudes, and practices of Ugandan athletes’: a cross-sectional study. Subst Abuse Treat Prev Policy. (2015) 10:37. [doi: 10.1186/s13011-015-0033-2](https://doi.org/10.1186/s13011-015-0033-2)

# Ozkan O, Torgutalp SS, Kara OS, Donmez G, Demirel H, Karanfil Y, et al. Doping knowledge and attitudes of turkish athletes: a cross-sectional study. Monten J Sports Sci Med. (2020) 9(1):49–55. [doi: 10.26773/mjssm.200307](https://doi.org/10.26773/mjssm.200307)

# Allen H, Price OJ, Hull JH, Backhouse SH. Asthma medication in athletes: a qualitative investigation of adherence, avoidance and misuse in competitive sport. J Asthma. (2022) 59(4):811–22. [doi: 10.1080/02770903.2021.1881968](https://doi.org/10.1080/02770903.2021.1881968)

# Antić D, Ostojić S. Evaluation of the information level on doping with high level Serbian athletes. Gazz Med Ital Arch Sci Med. (2012) 171(4):425–36.

# Azizi M, Mali AD, Tabari E. Study of prevalence of supplement use and knowledge of men national team rowers about doping and side effects. World Appl Sci J. (2012) 17(6):724–8.

# Ozdoğan Y, Ozcelik AO. Evaluation of the nutrition knowledge of sports department students of universities. J Int Soc Sports Nutr. (2011) 8(1):11. [doi: 10. 1186/1550-2783-8-11](https://doi.org/10.1186/1550-2783-8-11)

# Chan S, Lim M, Shamsuddin A, Mahmood T. Knowledge, attitude and perception of Malaysian pharmacy students towards doping in sports. J Pharm Pract Res. (2019) 49:135–41. [doi: 10.1002/jppr.1478](https://doi.org/10.1002/jppr.1478)

# Awaisu A, Mottram D, Rahhal A, Alemrayat B, Ahmed A, Stuart M, et al. Knowledge and perceptions of pharmacy students in Qatar on anti-doping in sports and on sports pharmacy in undergraduate curricula. Am J Pharm Educ. (2015) 79(8):119. [doi: 10.5688/ajpe798119](https://doi.org/10.5688/ajpe798119)

# Brown AA, Asare Pereko KK, Eliason S. Drug use in Ghana: knowledge, perceptions, and attitudes in a small group of elite student sportspersons. Biomed Hum Kinet. (2013) 5(1):1–5. [doi: 10.2478/bhk-2013-0001](https://doi.org/10.2478/bhk-2013-0001)

# Rintaugu EG, Mwangi FM. Knowledge, attitudes and perceptions on doping among university students in physical education and sport science related degree programmes. J Hum Sport Exerc. (2021) 16(1):174–86. [doi: 10.14198/jhse.2021.161.16](https://doi.org/10.14198/jhse.2021.161.16)

# Backhouse SH, McKenna J. Doping in sport: a review of medical practitioners’ knowledge, attitudes and beliefs. Int J Drug Policy. (2011) 22(3):198–202. [doi: 10. 1016/j.drugpo.2011.03.002](https://doi.org/10.1016/j.drugpo.2011.03.002)

# Murofushi Y, Kawata Y, Nakamura M, Yamaguchi S, Sunamoto S, Fukamachi H, et al. Assessing the need to use sport supplements: the mediating role of sports supplement beliefs. Perform Enhanc Health. (2024) 12(1):100269. [doi: 10.1016/j.peh.2023.100269](https://doi.org/10.1016/j.peh.2023.100269)

# Zia W, Javed A. Doping knowledge, beliefs and practices among physiotherapists. Khyber Med Univ J. (2020) 12. [doi: 10.35845/kmuj.2020.19718](https://doi.org/10.35845/kmuj.2020.19718)

# Mottram D, Khalifa S, Alemrayat B, Rahhal A, Ahmed A, Stuart M, et al. Perspective of pharmacists in Qatar regarding doping and anti-doping in sports. J Sports Med Phys Fitness. (2016) 56(6):817–24.

# Gebregergs Hailu H, Yirsaw Gobezie M, Tuha A, Mulugeta R, Ahmed Mohammed S. Doping knowledge, attitude and practice of pharmacists in dessie, Northeast Ethiopia. Integr Pharm Res Pract. (2021) 10:43–50. [doi: 10.2147/IPRP.S311204](https://doi.org/10.2147/IPRP.S311204)

# Blank C, Muller D, Schobersberger W. Discrepancy between knowledge and interest of Austrian sports physicians with respect to doping and doping prevention in sports. Int Sport Med J. (2014) 15(2):136–45.

# Jural LA, Soares TRC, Coqueiro RD, Rabello TB, Pithon MM, Maia LC. Development, validation and application of a Brazilian knowledge scale about sports doping in dentistry. Braz Oral Res. (2021) 35:e110. [doi: 10.1590/1807-3107bor-2021.vol35.0110](https://doi.org/10.1590/1807-3107bor-2021.vol35.0110)

# Kannan S, Naha A, Singh RR, Bansal P, Nayak VC, Goud S, et al. A descriptive cross-sectional study of knowledge of doping at grassroot level in India. Res J Pharm Technol. (2021) 14(12):6587–91. [doi: 10.52711/0974-360X.2021.01139](https://doi.org/10.52711/0974-360X.2021.01139)

# Lemettilä M, Leppä E, Pohjanoksa-Mäntylä M, Simula A, Koskelo J. Anti-doping knowledge and educational needs of Finnish pharmacists. Perform Enhanc Health. (2021) 9(2). [doi: 10.1016/j.peh.2021.100195](https://doi.org/10.1016/j.peh.2021.100195)

# Aly SM, Taha MA. Doping among physical education students: early-onset educational curriculum to counteract surrounding effect. Gazz Med Ital Arch Sci Med. (2020) 179(1-2):39–46. [doi: 10.23736/S0393-3660.18.04013-5](https://doi.org/10.23736/S0393-3660.18.04013-5)

# Blank C, Leichtfried V, Fürhapter C, Müller D, Schobersberger W. Doping in sports: west-Austrian sport Teachers’ and Coaches’ knowledge, attitude and behavior. Doping Sport Wissen Einst Verhalten West Österreichischer Sportlehrer Trainer. (2014) 65(10):289–93. [doi: 10.5960/dzsm.2014.133](https://doi.org/10.5960/dzsm.2014.133)

# Fürhapter C, Blank C, Leichtfried V, Mair-Raggautz M, Müller D, Schobersberger W. Evaluation of west-Austrian junior athletes’ knowledge regarding doping in sports. Wien Klin Wochenschr. (2013) 125(1-2):41–9. [doi: 10.1007/s00508-012-0318-7](https://doi.org/10.1007/s00508-012-0318-7)

# Lee YC, Chen CY, Cheng YY, Hsu MC, Chen TT, Chang WC. Assessing antidoping knowledge among Taiwanese pharmacists. BMC Med Educ. (2023) 23(1):808. [doi: 10.1186/s12909-023-04795-z](https://doi.org/10.1186/s12909-023-04795-z)

# Morente-Sánchez J, Zandonai T, Díaz MZ. Attitudes, beliefs and knowledge related to doping in different categories of football players. J Sci Med Sport. (2019) 22(9):981–6. [doi: 10.1016/j.jsams.2019.05.010](https://doi.org/10.1016/j.jsams.2019.05.010)

# Orr R, Grassmayr M, Macniven R, Grunseit A, Halaki M, Bauman A. Australian Athletes’ knowledge of the WADA prohibited substances list and performance enhancing substances. Int J Drug Policy. (2018) 56:40–5. [doi: 10.1016/j.drugpo.2018.02.025](https://doi.org/10.1016/j.drugpo.2018.02.025)

# Stojanovic G, Blagojevic MV, Bahtijari Z, Stankovic B, Markovic DT, Stojanovic D, et al. Analysis of knowledge and attitudes of the students of health and professional studies regarding the use of stimulative substances in sports. Vojnosanit Pregl. (2019) 76(2):144–51. [doi: 10.2298/VSP170214078S](https://doi.org/10.2298/VSP170214078S)

# Daniel M. Knowledge, attitude and practice on drug abuse among sports men and women in Lagos state, Nigeria. Int J Med Med Sci. (2012) 2:77–85.

# Dincer N, Demir H. The determination of knowledge levels related to doping in elite athletes. Turk J Sport Exerc. (2013) 15(2):56–69.

# Kedir HM, Kemal HS, Mekonen ZT, Huluka SA, Abiye AA. Pharmacy professionals’ knowledge, attitude and practice towards doping in sports: a cross-sectional study in Ethiopia. Heliyon. (2023) 9(11):e21207. [doi: 10.1016/j.heliyon.2023.e21207](https://doi.org/10.1016/j.heliyon.2023.e21207)

# Kim T, Kim YH. Korean National athletes’ knowledge, practices, and attitudes of doping: a cross-sectional study. Subst Abuse Treat Prev Policy. (2017) 12:7. [doi: 10.1186/s13011-017-0092-7](https://doi.org/10.1186/s13011-017-0092-7)

# Nyawose S, Naidoo R, Naumovski N, McKune AJ. Dietary supplements and beverages: knowledge, attitudes, and practices among semi-professional soccer players in KwaZulu-Natal, South Africa. S Afr J Sports Med. (2022) 34(1): v34i1a14018. [doi: 10.17159/2078-516x/2022/v34i1a14018](https://doi.org/10.17159/2078-516X/2022/v34i1a14018)

# Rotich JK, Rintaugu EG, Thangu E. Anti-doping knowledge, attitude, and experience of general practitioners in Kenya. J Sports Sci Res Spor Bil Araş Derg. (2023) 8(1):79–98. [doi: 10.25307/jssr.1200795](https://doi.org/10.25307/jssr.1200795)

# Tsivitanidou O, Christodoulides E, Petrou M. High-School Athletes’ knowledge, attitudes, and perceptions on doping: the cyprus sport-school study. Youth. (2023) 3:596–622. [doi: 10.3390/youth3020040](https://doi.org/10.3390/youth3020040)

# Ercan S, Oğul A, Canbulut A, Arslan E, Cetin C. Knowledge and attitudes of intern physicians on nutritional ergogenic supplementation and doping. Turk J Sports Med. (2021) 56. [doi: 10.47447/tjsm.0487](https://doi.org/10.47447/tjsm.0487)

# Königstein K, Gatterer K, Weber K, Schmidt-Trucksäss A, Tercier S, Blank C. Geographical heterogeneity of doping-related knowledge, beliefs and attitude among 533 youth olympics participants. J Sci Med Sport. (2021) 24(11):1116–22. [doi: 10.1016/j.jsams.2021.06.001](https://doi.org/10.1016/j.jsams.2021.06.001)

# Loraschi A, Galli N, Cosentino M. Dietary supplement and drug use and doping knowledge and attitudes in Italian young elite cyclists. Clin J Sport Med. (2014) 24(3):238–44. [doi: 10.1097/jsm.0000000000000018](https://doi.org/10.1097/JSM.0000000000000018)

# Morente-Sánchez J, Zabala M. Knowledge, attitudes and beliefs of technical staff towards doping in Spanish football. J Sports Sci. (2015) 33(12):1267–75. [doi: 10.1080/02640414.2014.999699](https://doi.org/10.1080/02640414.2014.999699)

# Yee KC, De Marco M, Salahudeen MS, Peterson GM, Thomas J, Naunton M, et al. Pharmacists as a source of advice on medication use for athletes. Pharmacy. (2020) 8(1):10. [doi: 10.3390/pharmacy8010010](https://doi.org/10.3390/pharmacy8010010)

# Ćorluka M, Gabrilo G, Blažević M. Doping factors, knowledge and attitudes among bosnian and herzegovinian football players. Dejavniki Dopinga Znanje Odnos Dopinga Med Nogometaši Bosne Hercegovine. (2011) 17(3):49–59.

# Engelberg T, Moston S, Blank C. Coaches’ awareness of doping practices and knowledge about anti-doping control systems in elite sport. Drugs Educ Prev Policy. (2019) 26(1):97–103. [doi: 10.1080/09687637.2017.1337724](https://doi.org/10.1080/09687637.2017.1337724)

# Farzin H, Esteghamati A, Razzaghi A, Nouri A. 2-how the Iranian free=style wrestlers know and think about doping?—a knowledge and attitude study. World Acad Sci Eng Technol. (2011) 5:209–14.

# Krishnan A, Datta K, Sharma D, Das Sharma S, Mahajan U, Jhajharia S, et al. Survey of antidoping knowledge, attitudes and practices amongst elite Indian sportsmen and the way forward. Med J Armed Forces India. (2022) 78(1):88–93. [doi: 10.1016/j.mjafi.2020.03.020](https://doi.org/10.1016/j.mjafi.2020.03.020)

# Mazanov J, Backhouse S, Connor J, Hemphill D, Quirk F. Athlete support personnel and anti-doping: knowledge, attitudes, and ethical stance. Scand J Med Sci Sports. (2014) 24(5):846–56. [doi: 10.1111/sms.12084](https://doi.org/10.1111/sms.12084)

# Murofushi Y, Kamihigashi E, Kawata Y, Yamaguchi S, Nakamura M, Fukamachi H, et al. The association between subjective anti-doping knowledge and objective knowledge among Japanese university athletes: a cross-sectional study. Front Sports Act Living. (2023) 5:1210390. [doi: 10.3389/fspor.2023.1210390](https://doi.org/10.3389/fspor.2023.1210390)

# Pöppel K, Büsch D. The doping critical attitude of elite sports coaches in combat sports. Ger J Exerc Sport Res. (2019) 49(2):168–78. [doi: 10.1007/s12662-019-00565-6 96](https://doi.org/10.1007/s12662-019-00565-6)

# Seif Barghi T, Halabchi F, Dvorak J, Hosseinnejad H. How the Iranian football coaches and players know about doping? Asian J Sports Med. (2015) 6(2):e24392. [doi: 10.5812/asjsm.6(2)2015.24392](https://doi.org/10.5812/asjsm.6(2)2015.24392)

# Yalcin I, Çalık F, Ece C, Geri S, Şeker R, Yalçın S. Knowledge levels and preference reasons regarding the doping of U23 Turkish national team athletes. Prog Nutr. (2019) 21:391–7.

# Zmuda Palka M, Bigosińska M, Siwek M, Angelova-Igova B, Mucha DK. Doping in sport-attitudes of physical trainers students regarding the use of prohibited substances increasing performance. Int J Environ Res Public Health. (2023) 20(5). [doi: 10.3390/ijerph20054574](https://doi.org/10.3390/ijerph20054574)

# Greenbaum DH, McLachlan AJ, Roubin RH, Moles R, Chaar BB. Examining pharmacists’ anti-doping knowledge and skills in assisting athletes to avoid unintentional use of prohibited substances. Int J Pharm Pract. (2023) 31(3):290–7. [doi: 10.1093/ijpp/riad015](https://doi.org/10.1093/ijpp/riad015)

# Nolte K, Steyn B, Krüger P, Fletcher L. Doping in sport: attitudes, beliefs and knowledge of competitive high school athletes in Gauteng province. S Afr J Sports Med. (2014) 26. [doi: 10.7196/sajsm.542](https://doi.org/10.7196/sajsm.542)

# Sepriani R, Bafirman B, Mudjiran M, Gusril G, Syafrudin S, Bachtiar S. Athlete doping knowledge analysis: a case study of the 20th national sports week (PON) papua 2021 in Indonesia. Int J Hum Mov Sports Sci. (2022) 10:723–31. [doi: 10.13189/saj.2022.100413](https://doi.org/10.13189/saj.2022.100413)

# Sertbas K, Akdeniz H, Yilmaz A, Çalik F, Sentürk U. Evaluation of ergogenic matter and doping usage knowledge of Turkish national athletes. Sports Medi J Med Sportiva. (2015) 11(3):2591–600.

# Uslu S, Barak R, Misovski A, Milenkoski J, Barak I, Işgüzar MG. Examination of elite volleyball players’ doping knowledge levels and their opinions on doping. Res Phys Educ Sport Health. (2020) 9(1):19–27. [doi: 10.46733/PESH2090019u](https://doi.org/10.46733/PESH2090019u)

# Yıldız Ö. The views of elite bodybuilding athletes concerning doping training, their level of knowledge about doping, and values education in sport. World J Educ. (2019) 9:56. [doi: 10.5430/wje.v9n1p56](https://doi.org/10.5430/wje.v9n1p56)

# Antic D. Evaluation of knowledge on doping in sports among serbian general practitioners. Med Pregl. (2017) LXX:25–31. [doi: 10.2298/MPNS1702025A](https://doi.org/10.2298/MPNS1702025A)

# Duda H, Stula A. Assessment of knowledge and attitude in the field of doping in young athletes of team games. Cent Eur J Sport Sci Med. (2022) 37(1):57–63. [doi: 10.18276/cej.2022.1-05](https://doi.org/10.18276/cej.2022.1-05)

# Starzak D, Derman W, McKune A, Semple S. Anti-doping knowledge and opinions of South African pharmacists and general practitioners. J Sports Med Doping Stud. (2016) 6:1–7. [doi: 10.4172/2161-0673.1000181](https://doi.org/10.4172/2161-0673.1000181)

# Woods CB, Moynihan A. General practitioners knowledge, practice and training requirements in relation to doping in sport. Ir Med J. (2009) 102(1):8–10.

# Pavlović R, Idrizović K. Attitudes of students of physical education and sports about doping in sport. Stavovi Studenata Fizičkog Vaspitanja I Sporta O Dopingu U Sportu. (2013) 11(1):103–13.
